# USP12 promotes antiviral responses by deubiquitinating and stabilizing IFI16

**DOI:** 10.1371/journal.ppat.1011480

**Published:** 2023-07-06

**Authors:** Yuling Fu, Xiaoxia Zhan, Xiaolong You, Dingnai Nie, Haiyan Mai, Yitian Chen, Shitong He, Junli Sheng, Zhijie Zeng, Hongwei Li, Jinlong Li, Shengfeng Hu

**Affiliations:** 1 Institute of Biotherapy, School of Laboratory Medicine and Biotechnology, Southern Medical University, Guangzhou, China; 2 Department of Laboratory Medicine, The First Affiliated Hospital of Sun Yat-sen University, Guangzhou, China; 3 Institute of Molecular Immunology, School of Laboratory Medicine and Biotechnology, Southern Medical University, Guangzhou, China; 4 The Second Affiliated Hospital, The State Key Laboratory of Respiratory Disease, Guangdong Provincial Key Laboratory of Allergy & Clinical Immunology, Guangzhou Medical University, Guangzhou, China; 5 Department of Rheumatology and Clinical Immunology, Zhujiang Hospital, Southern Medical University, Guangzhou, China; University of Southern California, UNITED STATES

## Abstract

Deubiquitinating enzymes (DUBs) regulate antiviral immune response through targeting DNA sensor signaling pathway members. As one of the DNA sensors, interferon (IFN)-γ inducible protein 16 (IFI16) play a major role in response to virus infections through activating the canonical STING/TBK-1/IRF3 signaling pathway. Only a few studies discuss the function of DUBs in IFI16-mediated antiviral response. Ubiquitin-specific protease 12 (USP12), which is one of the major members of the USP family, participates in various biological functions. However, whether USP12 regulates the nucleic acid sensor to modulate antiviral immune responses has not yet been elucidated. In this study, we found that knockout or knockdown of USP12 impaired the HSV-1-induced expressions of IFN-β, CCL-5, IL-6, and downstream interferon-stimulated genes (ISGs). Moreover, USP12 deficiency increased HSV-1 replication and host susceptibility to HSV-1 infection. Mechanistically, USP12 inhibited the proteasome-dependent degradation of IFI16 through its deubiquitinase activity, thereby maintaining IFI16 stability and promoting IFI16-STING-IRF3- and p65-mediated antiviral signaling. Overall, our findings demonstrate an essential role of USP12 in DNA-sensing signaling and contribute to the understanding of deubiquitination-mediated regulation of innate antiviral responses.

## Author summary

Ubiquitin-specific peptidase 12 (USP12), one of the major members of the deubiquitinating enzyme family, participates in various biological functions, such as cell proliferation and differentiation, apoptosis, neurodegeneration, tumor promoting, and antiviral immune responses. In this study, we reported that USP12 promoted IFI16-mediated innate antiviral signaling. Knockout or knockdown of USP12 impaired the DNA virus-triggered activation of the IFI16-STING-IRF3/NF-κB pathway and expression of downstream genes. USP12 deficiency increases HSV-1 replication and host susceptibility to HSV-1 infection. Mechanistically, USP12 inhibited the proteasome-dependent degradation of IFI16 through its deubiquitinase activity. Our results promote understanding of varied regulation of USP12 in antiviral signaling.

## Introduction

Innate antiviral immunity is the first line of host defense against viral infection. Immune cells activate antiviral responses upon recognition of pathogen-associated molecular patterns (PAMPs) by pattern recognition receptors (PRRs). After infection, the effective interaction between PAMPs and PRRs initiates a series of signaling cascades, which ultimately leads to the secretion of type I interferons (IFN-I), chemokines, and proinflammatory cytokines against viral pathogens [1]. Moreover, IFN-I subsequently induces interferon-stimulated genes (ISGs), such as ISG1, MX1, and IFIT1, which prevent viral entry and replication [2]. The recognition of viral nucleic acids involves three classes of PRRs: Toll-like receptors (primary TLR9), RNA sensors (primary RIG-I/MDA5), and DNA sensors. DNA sensors, including IFI16, DDX41, and cyclic GMP-AMP (cGAMP) synthase (cGAS), play a major role in response to virus infections through activating the canonical STING/TBK-1/IRF3 signaling pathway [3].

IFI16 regulates multiple cellular processes, including cell growth and differentiation [4], DNA damage response [5], apoptosis and senescence [6]. Unlike other DNA sensors, IFI16 can recognize both viral RNA and DNA in the nucleus and cytoplasm [7,8]. Studies suggest that IFI16 plays a vital role in antiviral immunity against HSV-1 [9], KSHV [10], HCMV [11], HPV [12] and HIV [13] infection. After detecting viral DNA or RNA, IFI16 induces the STING-dependent signal transduction through the TBK1-mediated IRF3 axis or IKKs-mediated NF-κB axis, which results in the production of IFN-I, proinflammatory cytokines, and chemokines against viral infection. However, innate antiviral immunity is strictly regulated as excessive immune response is harmful to the host.

Post-translational modifications, such as ubiquitination, are essential in regulating nucleic acid sensing and immune responses [14]. Ubiquitination is a dynamic and reversible process regulated through deubiquitination, which is mediated by deubiquitinating enzymes (DUBs). Studies have demonstrated the critical role of DUBs in regulating antiviral immune response through targeting the TLR-, RLR-, and cGAS-STING- mediated signaling cascades [15]. However, only a few studies discuss the function of DUBs in the IFI16-mediated antiviral response.

USP12, which is one of the major members of the ubiquitin-specific peptidase (USP) family, participates in various biological functions, such as cell proliferation and differentiation [16], apoptosis [17], neurodegeneration [18], and tumor-promoting [19]. We have recently reported that USP12 plays an intrinsic role in promoting the differentiation, activation, and proliferation of CD4^+^ T cells via the NF-κB signaling pathway through the deubiquitination and stabilization of B cell lymphoma/leukemia protein [20]. Notably, USP12 positively regulates interferon antiviral signaling independent of its deubiquitinase activity. Mechanistically, USP12 translocates from the cytoplasm to the nucleus and blocks the CREB-binding protein (CBP)-induced acetylation of phosphorylated STAT1 (p-STAT1), thereby maintaining the nuclear p-STAT1 levels and IFN antiviral activity [21]. However, it is unclear whether USP12 regulates nucleic acid sensors to modulate antiviral immune responses.

In this study, we demonstrated that during DNA virus infection, USP12 positively regulates the IFI16-STING-mediated antiviral response through its deubiquitinase activity. Results showed that USP12 deubiquitinated and stabilized IFI16, which regulated the IFI16-STING-TBK1-IRF3- and NF-κB- mediated antiviral response. Notably, the USP12 regulation of IFI16 had no effect on RNA virus infection.

## Results

### USP12 deficiency attenuated antiviral responses

**Fig 1:**
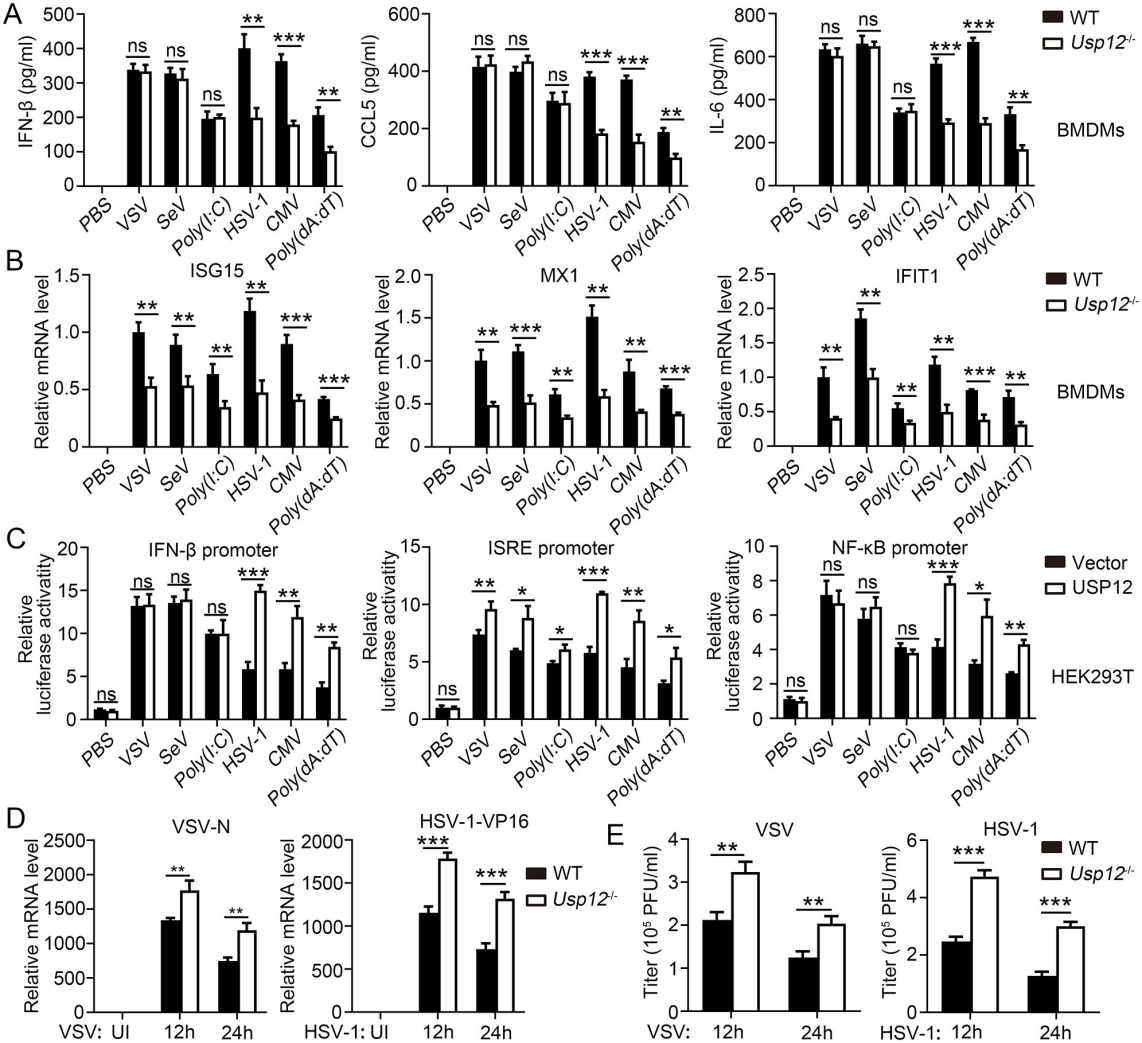
USP12 deficiency reduced the anti-virus response in BMDMs. WT or *Usp12*^-/-^ BMDMs were infected with VSV, SEV, HSV-1 or CMV, or transfected with Poly (I:C) or Poly(dA:dT) for 24 house or the indicated time. **(A)** ELISA analysis of IFN-β, CCL5 and IL-6 in the supernatants. **(B)** The expression of ISG15, MX1 and IFIT1 was analyzed by qPCR. **(C)** HEK293T cells transfected with USP12 or control plasmids (vector) were infected with infected with VSV, SEV, HSV-1 or CMV, or transfection with Poly (I:C) or Poly(dA:dT) for 24 house. Luciferase assay was performed to analyze regulation of IFN-β, NF-κB and MXI promoter by USP12. **(D)** Viral VSV-N RNA (left) and HSP-1-V16 RNA (right) were determined by qPCR. **(E)** Viral titres of VSV or HSV-1 virus were determined. Data shown are the mean ±SD. *P < 0.05, **P < 0.01 and ***P < 0.001. Ns, no significant. Data are representative of three independent experiments with similar results.

To explore the potential role of USP12 in antiviral immunity, we first detected the expression of USP12 after virus infection and found that USP12 expression was induced by HSV-1 infection (S1A and S1B Fig), which was mainly regulated by NF-κB p65 and IRF3, but not by STAT1 (S1C and S1D Fig). Next, we challenged USP12-deficient bone marrow-derived macrophages (BMDMs) or MEFs with the RNA virus, VSV or SEV, double-stranded RNA analog, poly(I:C), DNA virus, HSV-1 or CMV, or double-stranded DNA analog, poly(dA:dT) and analyzed the corresponding cytokine expressions. Results show that USP12 deficiency significantly inhibited IFN-β, CXCL5, and IL-6 production in BMDMs and MEFs infected with a DNA virus, although not in BMDMs infected with an RNA virus (Figs 1A and S2A). USP12 deficiency also significantly inhibited the expression of ISGs (ISG15, MX1, and IFIT1) in BMDMs and MEFs infected with a DNA or an RNA virus (Figs 1B and S2B). The exogenous overexpression of USP12 in HEK293T cells promoted DNA-virus induced luciferase activities of IFN-β, ISRE, and NF-κB promoters (Fig 1C). By contrast, overexpression of USP12 in the RNA virus infected-groups induced the ISRE promoter-driven luciferase activity (Fig 1C). Consistently, the RNA levels (Figs 1D and S2C) and viral titers (Figs 1E and S2D) of VSV and HSV-1 drastically increased replication in USP12-deficient BMDMs or MEFs.

**Fig 2:**
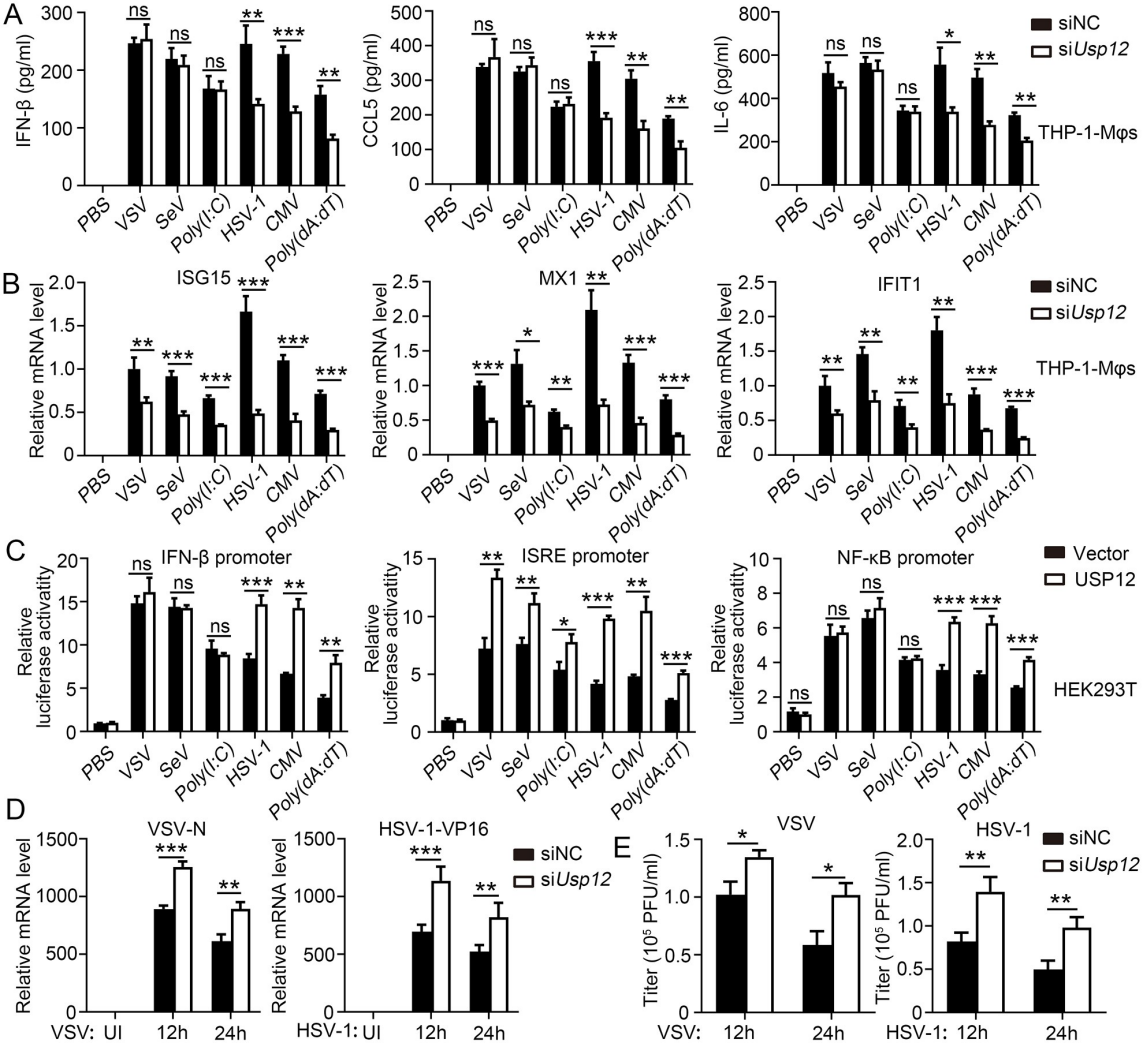
Knock down of USP12 reduced the anti-virus response in THP-1-Mφ. THP-1-induced macrophages (THP-1-Mφ) were infected with VSV, SEV, HSV-1 or CMV, or transfected with Poly (I:C) or Poly(dA:dT) for 24 hours or the indicated time. **(A)** ELISA analysis of IFN-β, CCL5 and IL-6 in the supernatants. **(B)** The expression of ISG15, MX1 and IFIT1 was analyzed by qPCR. **(C)** HEK293T cells transfected with USP12 or control plasmids (vector) were infected with infected with VSV, SEV, HSV-1 or CMV, or transfection with Poly (I:C) or Poly(dA:dT) for 24 hours. Luciferase assay was performed to analyze regulation of IFN-β, NF-κB and MXI promoter by USP12. **(D)** Viral VSV-N RNA (left) and HSP-1-V16 RNA (right) were determined by qPCR. **(E)** Viral titres of VSV or HSV-1 virus were determined. Data shown are the mean ±SD. *P < 0.05, **P < 0.01 and ***P < 0.001. Ns, no significant. Data are representative of three independent experiments with similar results.

Next, we explored whether USP12 has a similar role in human macrophages. THP-1 cells were induced into macrophages (THP-1-Mφs) and transfected with USP12 siRNA to knock down USP12 expression (S3A and S3B Fig). Similarly, the knockdown of USP12 decreased IFN-β, CCL5, and IL-6 production in the DNA virus infected-groups but not in the RNA virus infected-group (Fig 2A). USP12 knockdown also reduced the expression of ISGs (ISG15, MX1, and IFIT1) in both the DNA and RNA virus infected-groups (Fig 2B). The overexpression of human USP12 promoted DNA virus-induced luciferase activities of IFN-β, ISRE, and NF-κB promoters. By contrast, the RNA virus infected-groups showed an increased ISRE promoter-driven luciferase activity (Fig 2C). USP12 knockdown increased the replication of VSV and HSV-1, as indicated by the RNA levels (Fig 2D) and viral titers (Fig 2E). Overall, these results indicated that USP12 promotes anti-DNA and anti-RNA virus responses through combined regulation of cytokine production and ISG expression but regulation of ISG expression alone, respectively.

### USP12 overexpression promoted antiviral responses

To validate the effect of USP12 on antiviral response, we reconstituted USP12 into *Usp12*^-/-^ BMDMs and overexpressed USP12 in THP-1-Mφs. The results showed that USP12 overexpression did not affect the production of IFN-β and IL-6 in VSV-infected BMDMs (S4A Fig). However, it promoted the production of IFN-β and IL-6 in HSV-1-infected BMDMs and completely rescued the production of Ifn-β and Il-6 in *Usp12*^-/-^ BMDMs (S4B Fig). The overexpression of USP12 in BMDMs promoted the expressions of ISG15 and MX1 in both VSV- and HSV-1-infected BMDMs (S4C and S4D Fig) and reduced the replication of VSV and HSV-1, as indicated by the RNA levels (S4E Fig) and viral titers (S4F Fig). Similarly, USP12 overexpression did not affect the production of IFN-β, CCL5, and IL-6 during VSV infection (S5A Fig) but promoted the production of IFN-β, CCL5, and IL-6 in response to HSV-1 infection in THP-1-Mφs (S5B Fig). Moreover, USP12 overexpression upregulated ISG (ISG15, MX1, and IFIT1) expression (S5C and S5D Fig) and significantly inhibited HSV-1 and VSV replication, as indicated by the RNA levels (S5E Fig) and viral titers (S5F Fig). These results demonstrated that USP12 promotes both RNA and DNA virus sensing processes and is required for antiviral response.

### USP12-deficient mice were susceptible to virus infection

**Fig 3:**
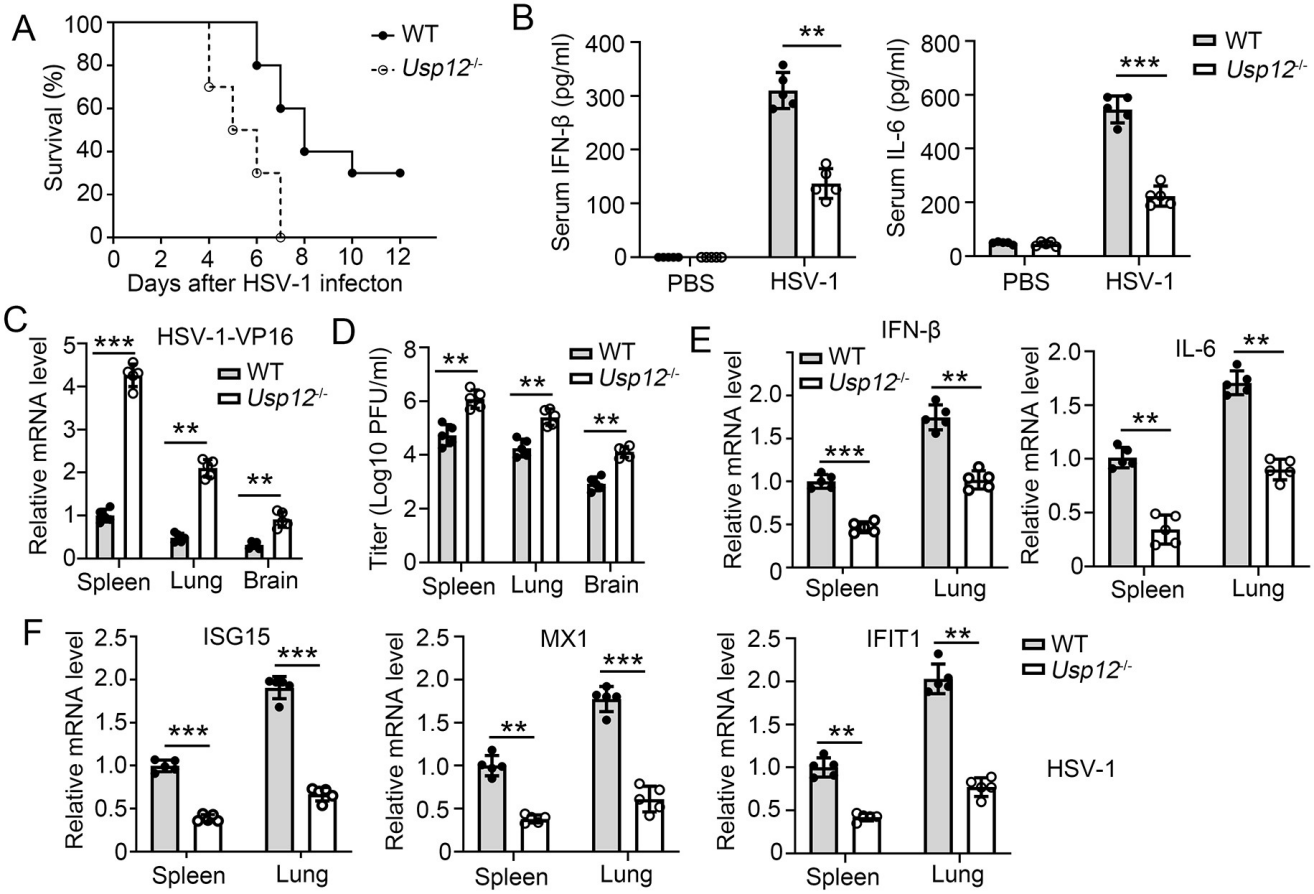
USP12-deficient mice were susceptible to HSV-1 infection. WT and *Usp12*^-/-^ mice were infected with HSV-1 virus (1 × 10^7^ PFU per mouse). **(A)** Survival rate of WT and *Usp12*^-/-^ mice after HSV-1 infection for indicated time. **(B)** Serum levels of IFN-β and IL-6 was determined by ELISA at 3 days post infection. **(C-E)** Spleen, lung and brain tissues were collected at 3 days post infection. **(C)** Viral HSP-1-VP16 mRNA were determined by qPCR. **(D)** Viral titres of VSV or HSV-1 virus were determined. **(E)** Expression of IFN-β and IL-6 was determined by qPCR. **(F)** Expression of ISG15, MX1 and IFIT1 was determined by qPCR. Data shown are the mean ±SD. *P < 0.05, **P < 0.01 and ***P < 0.001. Data are representative of three independent experiments with similar results.

To characterize the role of USP12 in virus infection *in vivo*, we infected wild type (WT) and *Usp12*^-/-^ mice with HSV-1 and VSV via tail vein injection and monitored their survival. The results showed that *Usp12*^-/-^ mice were more susceptible to lethal HSV-1 and VSV infection than WT mice (Figs 3A and S6A). Accordingly, decreased expressions of IFN-β and IL-6 were observed in the serum of *Usp12*^-/-^ mice with HSV-1 infection compared with those of mice with VSV infection (Figs 3B and S6B). The replication of HSV-1 and VSV exacerbated 4 d after viral infection in the lungs, spleens and brains of *Usp12*^-/-^ mice compared with that in the lungs and spleens of WT mice (Figs 3C, 3D and S6C). IFN-β and IL-6 expressions were severely impaired in the lungs, spleens and brains of *Usp12*^-/-^ mice during HSV-1 infection but not during VSV infection (Figs 3E and S6D). However, ISG (ISG15, MX1, and IFIT1) transcription was severely impaired in the lungs or spleens of *Usp12*^-/-^ mice infected with both HSV-1 and VSV (Figs 3F and S4E). Overall, these results demonstrated that Usp12 positively regulates the virus-induced expression of downstream genes and is essential for host defense against DNA and RNA viruses *in vivo*.

### USP12 targeted IFI16 to enhance antiviral response

**Fig 4:**
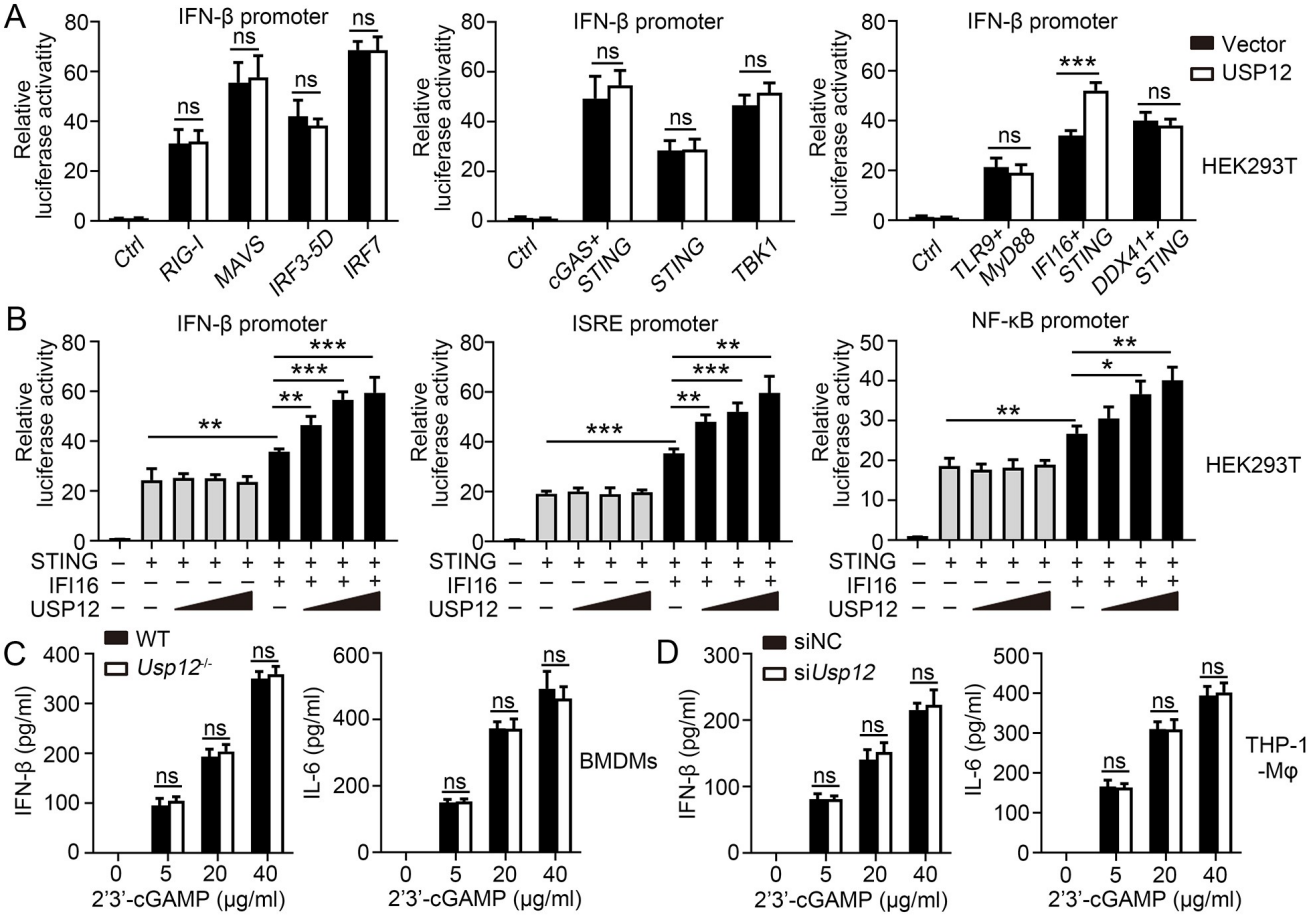
USP12 regulated IFI16-STING-mediated antiviral response by targeting at IFI16. **(A)** Luciferase reporter assays analyzing IFN-β promoter activity of HEK293 cells co-transfected with USP12 and indicated RNA/DNA sensors for 36 hours. RNA or DNA sensor and downstream pathway including RIG-1/MAVS/IRF3/IRF7, cGAS-STING-TBKI, TLR9/Myd88, IFI16/STING, and DDX41/STING. **(B)** Luciferase reporter assays analyzing IFN-β, ISRE and NF-κB promoter activity of HEK293T cells transfected with IFI16 or STING plasmids and increasing doses of expression vector for USP12. **(C)** WT and *Usp12*^-/-^ BMDM cells were transfected with increasing doses of cGAMP before undergoing ELISA analysis of the production of IFN-β and IL-6. (D)THP-1 Mφ transfected with control or USP12-specific siRNA were transfected with increasing doses of cGAMP. The production of IFN-β and IL-6 was determined by ELISA. Data shown are the mean ±SD. *P < 0.05, **P < 0.01 and ***P < 0.001. Ns, no significant. Data are representative of three independent experiments with similar results.

A previous study has demonstrated that USP12 regulates the IFN/JAK-mediated ISG expression by inhibiting the CBP acetyltransferase activity. However, the mechanism by which USP12 regulates IFN-β and IL-6 production during DNA virus infection remains unclear. As cytosolic DNA and RNA sensors play an essential role in IFN-I-mediated antiviral response, we performed luciferase reporter assays to screen for multiple DNA and RNA sensors that interact with USP12 in mediating IFN-β response. Consistently, USP12 did not affect the IFN-β promoter activity mediated by RIG-1, MAVS, IRF3-5D, and IRF7, which are the major molecules in a signaling pathway against RNA virus infection (Fig 4A). It also did not affect the IFN-β promoter activity mediated by cGAS/STING, STING, and TBK1, which are the major molecules in a vital signaling pathway against DNA virus infection (Fig 4A). Furthermore, USP12 significantly enhanced the IFN-β promoter activity mediated by IFI16/STING but not by TLR9/MYD88 and DDX41/STING (Fig 4A). In addition, luciferase reporter assay results showed that USP12 did not promote the IFN-β promoter activity in the absence of IFI16. By contrast, USP12 enhanced IFN-β, NF-κB, and ISRE promoter activities in a dose-dependent manner after cotransfection with IFI16 (Fig 4B). cGAMP is a key molecule that activates STING and induces an antiviral response and IFN-I secretion during DNA virus infection. Our results showed that Usp12 deficiency did not affect the production of Ifn-β and Il-6 in response to transfected cGAMP in BMDMs (Fig 4C) or THP-1-Mφs (Fig 4D). These results indicated that USP12 regulates the IFI16-STING mediated antiviral response by directly regulating IFI16. After STING activation, TBK1 is recruited and activated to induce the activation of downstream IRF3 and NF-κB. Further evaluations showed that USP12 deficiency decreased the phosphorylation of p65 and IRF3 induced by HSV-1 (S7A Fig) but not by VSV (S7B Fig). Overall, these experiments suggested that USP12 targets IFI16 to enhance antiviral response.

### USP12 deubiquitinated and stabilized IFI16

**Fig 5:**
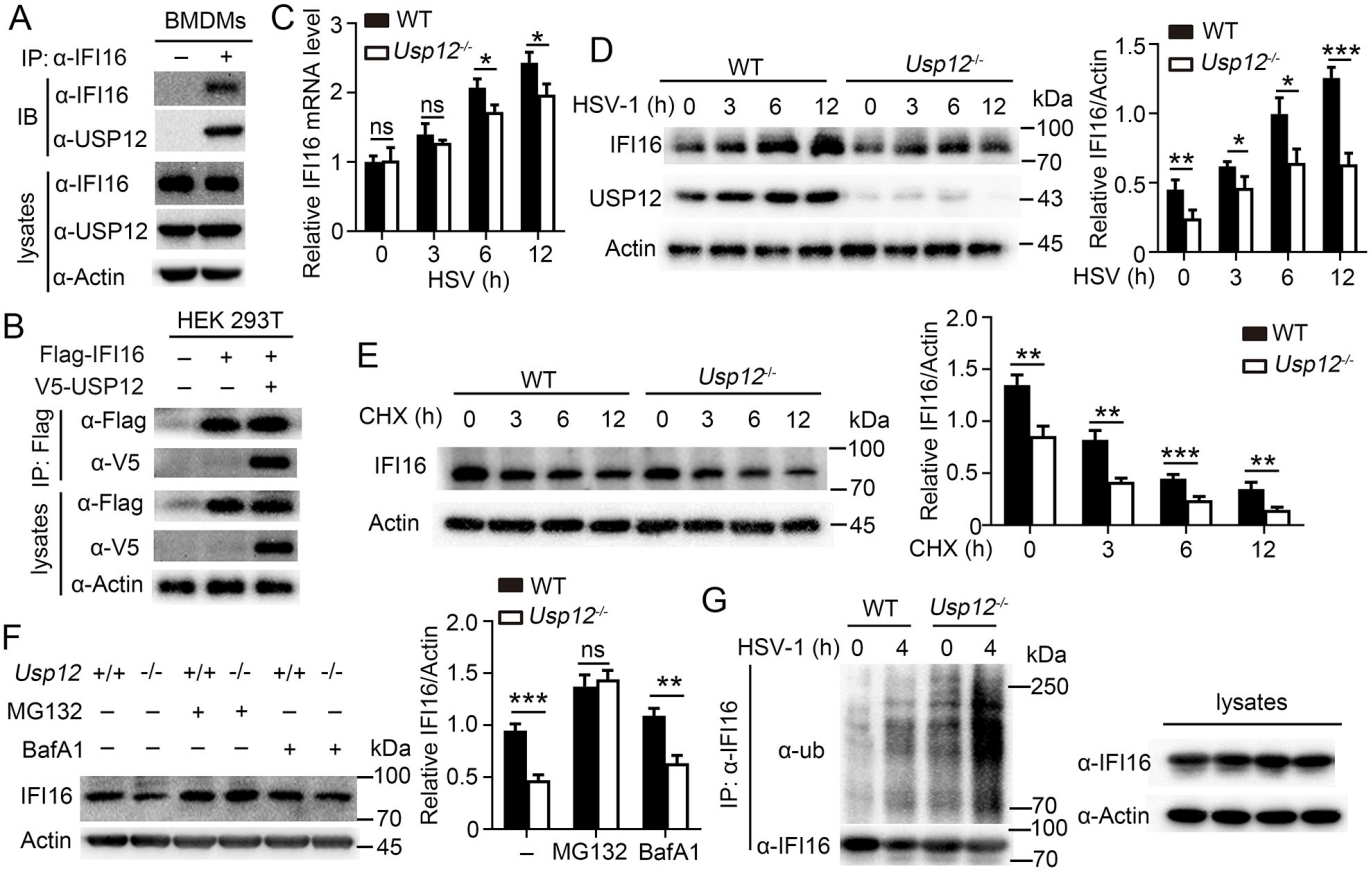
USP12 deubiquitinated and stabilized IFI16. **(A)** WT BMDMs were infected HSV-1 for 6 hours followed by immunoprecipitation (IP) using anti-IFI16 or IgG, and immunoblotting (IB) analysis. **(B)** IP and IB analysis of HEK293T cells that were transfected with plasmids encoding V5-USP12 and Flag-IFI16 for 24 hours. **(C-D)** WT and *Usp12*^-/-^ BMDMs were infected with HSV-1 for the indicated times. IFI16 expression levels were detected by qPCR **(C)** or western blot **(D)**. Densitometry quantification of band intensity are presented in the right panel. **(E)** WT and *Usp12*^-/-^ BMDMs were treated with cycloheximide (CHX, 100 μg/ml) for the indicated times before being analyzed by western blot with antibodies against IFI16. Densitometry quantification of band intensity are presented in the right panel. **(F)** WT and *Usp12*^-/-^ BMDMs were pretreated with MG132 or BafA1 for 4 hours, infected with HSV-1 for 6 hours, and harvested for subsequent western blot. Densitometry quantification of band intensity are presented in the right panel. **(G)** IFI16 IB and ubiquitination analysis using whole-cell extracts of WT and *Usp12*^-/-^ BMDMs infected with HSV-1 for the indicated time. Data shown are the mean ±SD. *P < 0.05, **P < 0.01 and ***P < 0.001. Ns, no significant. Data are representative of three independent experiments with similar results.

Immunoprecipitation and immunoblot assays were performed to validate whether USP12 interacts with IFI16. The results showed that the endogenous USP12 formed a stable complex with IFI16 in BMDMs (Fig 5A). Similarly, USP12 interacted with IFI16 in HEK293 cells (Fig 5B). USP12 has been shown to have very limited deubiquitylating activity in the absence of other adaptor proteins such as UAF1 (or WDR48) and WDR20[22]. We found that the endogenous USP12 formed a stable complex with UAF1 and WDR20 in BMDMs (S8A Fig). IFI16 mRNA expression did not differ at the early stages of infection but showed a significant difference in protein expression between WT and *Usp12*^*-/-*^ BMDMs stimulated with HSV-1 (Fig 5C and 5D), CMV (S8B and S8C Fig) or poly(dA:dT) (S8D and S8E Fig). UAF1 deficiency led to decreased IFI16 protein level (S8F Fig). Inhibition of STAT1 activity resulted in a significant decrease in IFI16 expression, while USP12 deficiency did not affect the mRNA levels of IFI16 but impacted its protein abundance (S8G and S8H Fig). Subsequently, we investigated whether USP12 regulates IFI16 level through its deubiquitinase activity. We found that USP12 deficiency promoted the degradation of IFI16 (Fig 5E). IFI16 degradation in *Usp12*^-/-^ BMDMs was completely inhibited by the proteasome inhibitor, MG132 (Fig 5F). The ubiquitination level of IFI16 was significantly increased in *Usp12*^-/-^ BMDMs compared with that in WT BMDMs (Fig 5G). Furthermore, we found that USP12 regulates the K48 ubiquitination of IFI16, but it does not exert any influence on its K63 ubiquitination (S8I Fig). These results demonstrated that USP12 deubiquitinates and stabilizes IFI16 during DNA virus infection.

### USP12 stabilized IFI16 dependent of its deubiquitinase activity

**Fig 6:**
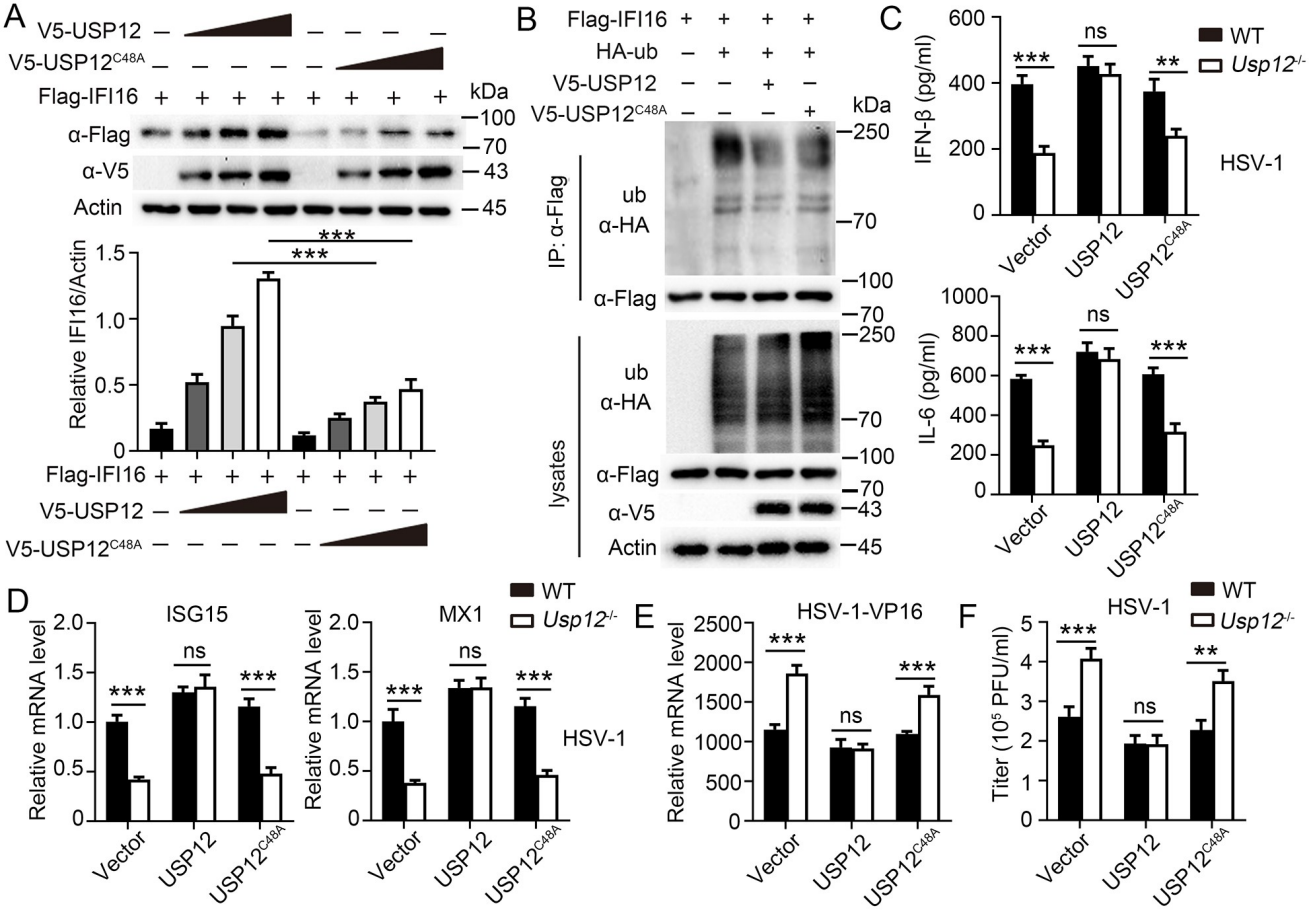
USP12 stabilized IFI16 dependent on its deubiquitive activity. **(A)** Western blot analysis of extracts of HEK293T cells transfected with IFI16-Flag vector and increasing doses of expression vector for V5-USP12 or V5-USP12^C48A^. Densitometry quantification of band intensity are presented in the below panel. **(B)** Immunoblot (IB) and ubiquitination analysis of extracts of HEK293T cells transfected with indicated plasmids. **(C-E)** WT or *Usp12*^-/-^ BMDMs were transfected with control or expression vector for V5-USP12 or V5-USP12^C48A^, and infected with HSV-1for 24h. **(C)** Production of IFN-β and IL-6 was determined by ELISA. **(D)** Expression of ISG15 and MX1 was determined by qPCR. **(E)** Viral HSP-1-V16 RNAs were determined by qPCR. **(F)** Viral titres were determined. Data shown are the mean ±SD. *P < 0.05, **P < 0.01 and ***P < 0.001. Ns, no significant. Data are representative of three independent experiments with similar results.

To investigate whether USP12 stabilized IFI16 based on its deubiquitinase activity, an enzymatically inactive mutant, USP12^C48A^, was developed. We found that USP12, but not USP12^C48A^, reduced the IFI16 degradation rate (Fig 6A) and ubiquitination level (Fig 6B) in HEK293T cells, which indicated that USP12 stabilized IFI16 and reversed IFI16 ubiquitination by its deubiquitinase activity. Furthermore, we reconstituted Usp12 or Usp12^C48A^ into WT and *Usp12*^-/-^ BMDMs and subjected them to HSV-1 infection. ELISA revealed that overexpression of Usp12, but not Usp12^C48A^, substantially restored the production of IFN-β and IL-6 in *Usp12*^-/-^ BMDMs (Fig 6C). The expression of ISGs (ISG15 and MX1) was also rescued in *Usp12*^-/-^ BMDMs reconstituted with Usp12 but not with Usp12^C48A^ (Fig 6D). In addition, RNA levels (Fig 6E) and viral titers (Fig 6F) showed that overexpression of Usp12, but not Usp12^C48A^, decreased HSV replication in *Usp12*^-/-^ BMDMs. Accordingly, increased HSV-1-induced expression of IFN-β, CCL-5, IL-6, and ISGs (ISG15, MX1, and IFIT1) (S9A and S9B Fig) and decreased HSV replication (S9C and S9D Fig) were observed in THP-1-Mφs reconstituted with USP12 but not USP12^C48A^. Overall, these data suggested that USP12 stabilizes IFI16 and regulates antiviral response based on its deubiquitinase activity.

### USP12-regulated antiviral response depends on Ifi16

**Fig 7:**
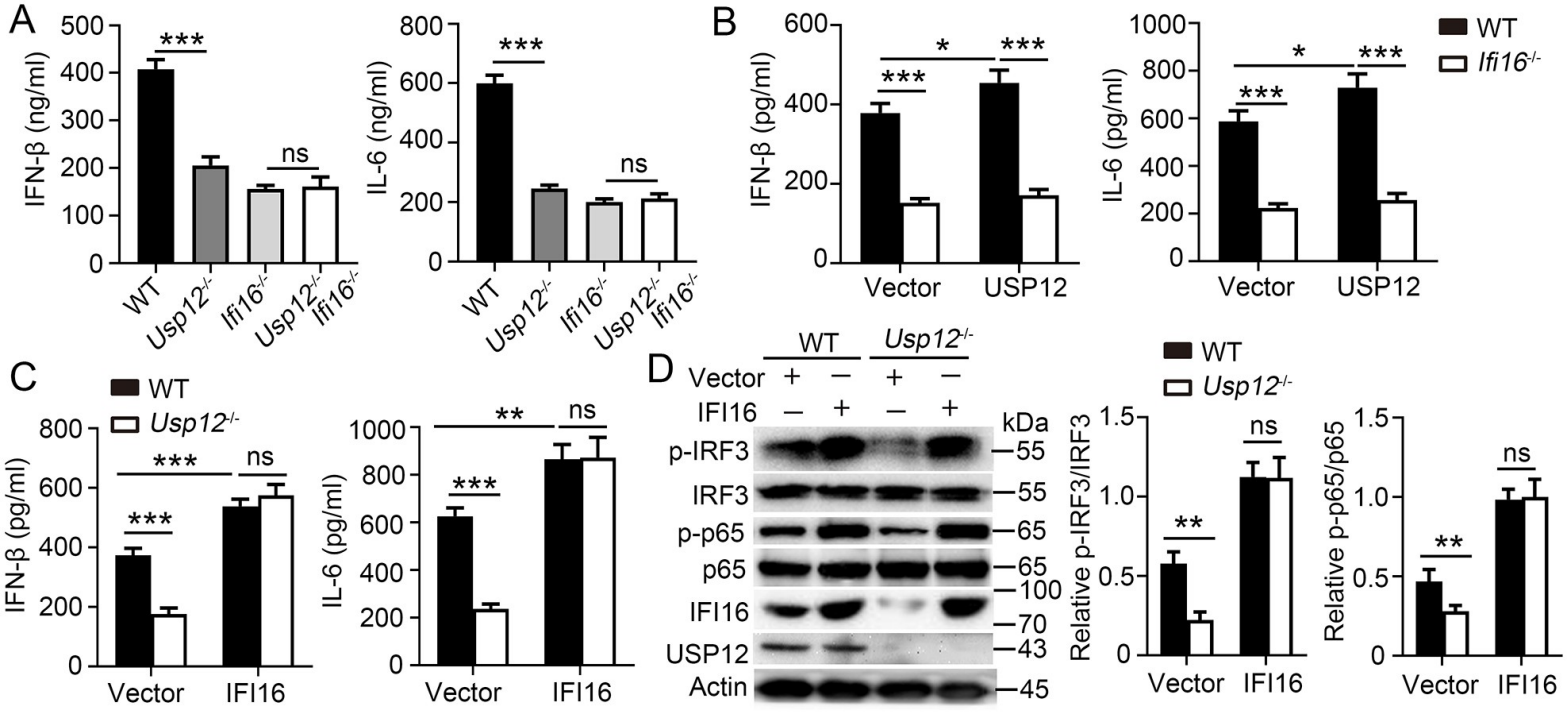
USP12 function dependent on IFI16. **(A)** ELISA analysis of IFN-β and IL-6 in the supernatants of WT, *Usp12*^-/-^, *Ifi16*^-/-^ and *Usp12*^-/-^*Ifi16*^-/-^ BMDMs infected with HSV-1 for 24 h. **(B)** ELISA analysis of IFN-β and IL-6 in the supernatants of WT or *Ifi16*^-/-^ BMDMs transfected with control, or expression vector for USP12. **(C)** ELISA analysis of IFN-β and IL-6 in the supernatants of WT or *Usp12*^-/-^ BMDMs transfected with control or expression vector for IFI16. **(D)** Western blot analysis of extracts of WT or *Usp12*^-/-^ BMDMs transfected with indicated vectors. Densitometry quantification of band intensity are presented in the right panel. Data shown are the mean ±SD. *P < 0.05, **P < 0.01 and ***P < 0.001. Ns, no significant. Data are representative of three independent experiments with similar results.

To assess whether IFI16 is the primary target of USP12 in regulating antiviral response, USP12 and IFI16 double knockout (*Usp12*^*-/-*^*Ifi16*^*-/-*^) mice were developed. The results showed no difference in the expressions of IFN-β and IL-6 between the two IFI16-deficient groups, irrespective of USP12 deficiency (Fig 7A). However, IFI16 deficiency did not rescue the reduced ISG expression caused by USP12 deficiency (S10A Fig). Furthermore, USP12, but not IFI16, deficiency significantly decreased the ISG expression induced by IFN-β in BMDMs (S10B Fig). Usp12 overexpression in *Ifi16*^-/-^ BMDMs did not rescue the reduced expression of IFN-β and IL-6 caused by Ifi16 deficiency (Fig 7B). By contrast, IFI6 overexpression in *Usp12*^-/-^ BMDMs promoted the expression of IFN-β and IL-6 (Fig 7C) and phosphorylation of IRF3 and p65 (Fig 7D). However, IFI16 overexpression in *Usp12*^-/-^ BMDMs did not rescue the reduced ISG expression due to USP12 deficiency (S10C Fig). These results demonstrated that USP12 regulates IFN-β and IL-6 production in an IFI16-dependent manner.

## Discussion

IFI16 plays a critical role as a DNA sensor that can recognize both pathogenic DNA and RNA. Unlike other DNA sensors, it can redistribute between the nucleus and cytoplasm; thus, it detects pathogenic DNA or RNA in a localization-dependent manner [23]. Although studies have shown that DUBs regulate antiviral immune response through targeting the TLR, RLR, and cGAS-STING signaling cascades [15], whether DUBs control IFI16 levels to mediate immune response against infection remains unknown. In this study, we found that USP12 significantly inhibited IFI16 ubiquitination and degradation, stabilized and extended the half-life of IFI16, and subsequently induced the STING-dependent expressions of IFN-β, CCL-5, IL-6, and downstream ISGs, which makes the host more resistant to DNA virus (S11 Fig).

USP12 participates in the development of various diseases, such as neurodegeneration [18], cancer [24,25] and inflammatory diseases [20,26]. More recently, the role of USP12 in antiviral response has gained increasing attention. USP12 knockdown by siRNAs promotes the replication of RGNNV, whereas USP12 overexpression increases the transcription level of inflammatory factors and interferon-related genes [27]. Moreover, USP12, which is a cell-intrinsic inhibitor of the acetyltransferase, CBP, translocates into the nucleus and regulates the nuclear-p-STAT1-mediated antiviral response independent of its deubiquitinase activity [21]. The study showed that USP12 does not regulate the production of IFN-β, but productions of ISGs during RNA virus infection, which is consistent with our results. However, unlike its role on anti-RNA viruses, we found that USP12 not only regulates the production of ISGs, but also regulates the expression of IFN-β during DNA virus infection. Our results showed that USP12 inhibited IFI16 ubiquitination and degradation through its deubiquitinase activity, thereby promoting IFI16-STING mediated antiviral response. Our results complemented previous reports that support the essential role of USP in antiviral response.

Post-translational modifications that influence the stability and location of IFI16 determine the outcome of an antiviral response. The function of post-transcriptional regulation of IFI16 in viral infection is diverse. Viruses evade immune surveillance through post-transcriptional regulation of IFI16. In particular, at the late stage of cytomegalovirus (HCMV) infection, IFI16 binds to the viral structural protein, PUL97, and is phosphorylated and mislocalized to the cytoplasmic virus assembly complex, which allows HCMV to escape the intracellular viral restriction [28]. Moreover, HPV recruits TRIM21 to mediate the ubiquitination and degradation of IFI16 inflammasome, which leads to the inhibition of cell pyroptosis and self-escape from immune surveillance [12]. Notably, the herpesvirus nuclear protein, ICP0, binds nuclear IFI16 and promotes the degradation of IFI16 to inhibit IFN-β expression, which leads to evasion of innate immune surveillance [9]. However, post-transcriptional regulation of IFI16 inhibits excessive inflammation. In particular, STING-TRIM21 attenuates antiviral immunity through promoting IFI16 ubiquitination and degradation to achieve a negative feedback regulation of IFI16 and control the overactivation of host immunity [29]. Moreover, our study has shown that the USP12-associated deubiquitination of IFI16 promoted an antiviral response. Various DUBs regulate members of the RNA or DNA sensing signaling pathways, such as MAVS, cGAS, DDXI, IFI16, and STING [30]. In particular, USP18 recruits USP20 to promote innate antiviral response by the deubiquitination and stabilization of STING [31]. In this study, USP12 had no effect on the c-GAS-STING, DDX41-STING, TLR-9-MYD88, and RIG-MDA-5-IRF3 signaling pathways. However, USP12 may positively regulate the IFI16-STING-mediated IFN-I antiviral response by deubiquitinating and stabilizing IFI16. Notably, USP12-mediated ISG response was independent of IFI16 as either IFI16 deficiency in BMDMs or overexpression in *Usp12*^-/-^ BMDMs had no impact on ISG transcription. USP12 indirectly regulates ISGs production by regulating IFN-β expression. In addition, as the nuclear USP12 inhibited the acetyltransferase activity of CBP and regulated the p-STAT1-mediated IFN antiviral response [21]. These findings promote the understanding of varied regulation of USP12 in antiviral signaling.

In summary, we reported that USP12 promoted IFI16-mediated innate antiviral signaling. Knockout or knockdown of USP12 impaired the DNA virus-triggered activation of the IFI16-STING-IRF3/NF-κB pathway and expression of downstream genes. USP12 deficiency increases HSV-1 replication and host susceptibility to HSV-1 infection. Mechanistically, USP12 inhibited the proteasome-dependent degradation of IFI16 through its deubiquitinase activity. Overall, these findings indicate that USP12 plays an essential role in innate antiviral immune responses.

## Materials and methods

### Ethics statement

All animal experiments were conducted in accordance with protocols approved by the Medical Ethics Board and the Biosafety Management Committee of Southern Medical University (SMU-L2020018). All mice were used at an age of 6–12 weeks and were randomly divided into different groups.

### Mice

C57BL/6 mice (Wild type, WT) were from the Lab Animal Center of Southern Medicine University (Guangzhou, China). The *Usp12*^-/-^, *Ifi16*^-/-^ and *Uaf*1^*fl/fl*^ (*Wdr48*^*fl/fl*^) mice on a C57BL/6J background were generated by Cyagen Biosciences Inc. (Guangzhou, China) using CRISPR-Pro technology. *Usp12*^-/-^ were crossed with *Ifi16*^-/-^mice to generate *Usp12*^-/-^*Ifi16*^-/-^mice. All mice were all C57BL/6 background and maintained in the Lab Animal Center of Southern Medicine University under specific pathogen-free conditions.

### Cell culture

BMDM, THP-1 cell line, human embryonic kidney cells (HEK293) and mouse embryonic fibroblasts (MEFs) were maintained in-house. Cells were cultured in DMEM supplemented with 10% fetal bovine serum, 1% streptomycin and penicillin. Primary BMDMs were produced as previously described [32]. Briefly, bone marrow cells were isolated from mouse femur, then cells were cultured in DMEM containing 10% fetal bovine serum, 1% streptomycin and penicillin, and 10 M β- mercaptoethanol, with GM-CSF (10 ng/ml, Peprotech) for BMDM differentiation. On day 6, the cells were re-seeded and cultured in 4-hydroxyltamoxifen-free medium for 24h before subsequent experiments.

### siRNA

The siRNAs were synthesized and transfected into THP-1-induce macrophages (THP-1-Mφ) using Lipofectamine 2000 according to the manufacturer’s manual. Twenty-four hours after transfection, cells were harvested or stimulated with virus followed by immunoblot, qPCR or ELISA.

### Viral infection

Cells were seeded into 24-well plates (2 × 10^5^ cells per well) or six-well plates (10^6^ to 10^7^ cells per well). Twenty-four hours later, cells were infected with SeV, VSV, HSV-1 or CMV (MOI = 1), or transfected with Poly (dA:dT) or Poly(I:C) for indicated time. Then cells were collected for qPCR or immunoblot assays, and the supernatants were saved for ELISA analysis. For mice infection, age- and sex-matched *Usp12*^-/-^ and WT mice were injected i.v. with VSV (1 × 10^7^ pfu/mouse) or HSV-1 (1 × 10^7^ pfu /mouse) and the survival of animals was monitored every day. The sera, lungs or spleen from infected mice were collected for qPCR or ELISA analysis at 12 or 24 h after infection, respectively. For virus replication assays, viruses were removed at 1 hour after infection. Cells were washed with PBS for twice followed by culture with full medium for the indicated time points.

### Enzyme-linked immunosorbent assay (ELISA)

Cytokine production in supernatants of *in vitro* cell cultures or sera of mice was measured by ELISA of mouse IFN-β, IL-6 and CCL5 (ExCell Bio, China) according to the manufacturer’s protocol.

### RNA isolation and qPCR

Total RNAs were extracted from different cells using TRIzol reagent (Invitrogen). The cDNA was produced by reverse transcription using oligo (dT), and then analyzed by quantitative real-time PCR (qPCR) using IFN-β, IL-6 and CCL5, ISG15, MX1, IFIT1, HSV-VP16, VSV-N, and SYBR GreenSupermix (Bio-Rad Laboratories). The primer sequences used for PCR are in Table A in S1 Text.

### Plaque assay

Homogenates of lung or spleen (or the serial dilutions) from infected WT or *Usp12*^-/-^mice were used to infect monolayers of Vero cells. One hour later, the homogenates or dilutions were removed and the infected Vero cells were washed with pre-warmed PBS twice followed by incubation with DMEM containing 2% methylcellulose for 48 h. Cells were fixed with 4% paraformaldehyde for 15 min and stained with 1% crystal violet for 30 min before counting the plaques.

### Immunoblot, co-immunoprecipitation and ubiquitination assays

The experiments were performed as previously described [33]. Equal amounts of cell lysates were resolved using 8±15% polyacrylamide gels transferred to PVDF membrane. Membranes were blocked in 5% non-fat dry milk in PBST and incubated overnight with the respective primary antibodies at 4°C. The membranes were incubated at room temperature for 1 h with appropriate HRP-conjugated secondary antibodies and visualized with Plus-ECL (PerkinElmer, CA) according to the manufacturer’s protocol. For immunoprecipitation assays, the lysates were immunoprecipitated with IgG or the appropriate antibodies and protein G Sepharose beads. The precipitates were washed three times with lysis buffer containing 500 mM NaCl, followed by immunoblot analysis. For deubiquitination assays, the cells were lysed with the lysis buffer and the supernatants were denatured at 95 °C for 5 min in the presence of 1% SDS. The denatured lysates were diluted with lysis buffer to reduce the concentration of SDS below 0.1% followed by immunoprecipitation with the indicated antibodies. The immunoprecipitates were subjected to immunoblot analysis with anti-ubiquitin chains. Antibodies used in this study are listed in Table B in S1 Text.

### Transfection and luciferase reporter assay

HEK293 cells were co-transfected with the IFN-β promoter, ISRE promoter and NF-kB promoter luciferase reporter plasmid and a TK-Renilla luciferase reporter, together with vector alone or USP12, USP12^C48A^, IFI16, RIG-I, MAVS, IRF3-5D, IRF7, cGAS, STING, TBK1, TLR9, MyD88, and DDX41 constructs. Twenty-four hours later, cells were infected with HSV or VSV for 6 h. Luciferase reporter activities were measured in triplicate using the Dual-Luciferase reporter assay system (Promega, Madison, WI, USA), according to the manufacturer’s protocol, and quantified using the 96-well plate luminometer (Promega). The firefly luciferase to Renilla luciferase ratios were determined and were defined as the relative luciferase activity.

### Lentivirus-mediated gene transfer

HEK293T cells were transfected with phage-6tag-USP12, phage-6tag-USP12^C48A^, phage-6tag-IFI16, or the empty vector along with the packaging vectors pSPAX2 and pMD2G. The medium was changed for fresh full medium (10% FBS, 1% streptomycin-penicillin and 10 μM β-mercaptoethanol) after 8 h. Forty hours later, supernatants were harvested to infect BMDMs or THP-1-Mφ followed by various analyses.

### Statistics

All experiments were performed at least thrice. When shown, multiple samples represent biological (not technical) replicates of mice randomly sorted into each experimental group. No blinding was performed during animal experiments. Determination of statistical differences was performed with Prism 8 (Graphpad Software, Inc.) using unpaired two-tailed t-tests (to compare two groups with similar variances), or two-way ANOVA with Bonferonni’s multiple comparison test (to compare more than two groups).

## Supporting information

S1 Fig Related to Fig 1. HSV-1 infection induced the expression of USP12 in BMDMs. **(A)** The mRNA expression of USP12 were assessed using qPCR analysis in WT mouse BMDMs infected with HSV-1 for indicated time. **(B)** The protein abundance of USP12 were assessed using western blot in BMDMs infected with infected with HSV-1 for indicated time. **(C-D)** WT BMDMs were pretreated with NF-κB p65 inhibitor JSH-23 (p65 i), IRF3 inhibitor Geldanamycin (IRF3 i), or STAT1 inhibitor Fludarabine (STAT1 i), and infected with HSV-1 for indicated time. **(C)** The mRNA expression of USP12 were assessed using qPCR. **(D)** The protein abundance of USP12 were assessed using western blot. Data shown are the mean ±SD. *P < 0.05, **P < 0.01 and ***P < 0.001. Ns, no significant. Data are representative of three independent experiments with similar results.
(TIF)

S2 Fig Related to Fig 1. USP12 deficiency reduced the anti-virus response in MEFs. WT or *Usp12*^-/-^ MEFs were infected with VSV, SEV, HSV-1 or CMV, or transfected with Poly (I:C) or Poly(dA:dT) for 24 house or the indicated time. **(A)** ELISA analysis of IFN-β, CCL5 and IL-6 in the supernatants. **(B)** The expression of ISG15, MX1 and IFIT1 was analyzed by qPCR. **(C)** Viral VSV-N RNA (left) and HSP-1-V16 RNA (right) were determined by qPCR. **(D)** Viral titres of VSV or HSV virus were determined. Data shown are the mean ±SD. *P < 0.05, **P < 0.01 and ***P < 0.001. Ns, no significant. Data are representative of three independent experiments with similar results.
(TIF)

S3 Fig Related to Fig 2. HSV-1 infection induced the expression of USP12 in THP-1-Mφs. **(A)** The mRNA expression of USP12 were assessed using qPCR analysis in THP-1-Mφs infected with HSV-1 for indicated time. **(B)** The protein abundance of USP12 were assessed using western blot in THP-1-Mφs infected with infected with HSV-1 for indicated time. Data shown are the mean ±SD. Data are representative of three independent experiments with similar results.
(TIF)

S4 Fig Overexpression of USP12 in BMDM promoted the anti-virus response. WT or *Usp12*^-/-^ BMDMs transfected with control or expression vector for USP12 were infected with VSV or HSV virus for 24 hours. Production of IFN-β and IL-6 was determined by ELISA after VSV infection **(A)** or HSV-1 infection **(B)**. QPCR was performed to analyze the expression of ISG15 and MX1 after VSV **(C)** and HSV **(D)** infection. **(E)** Viral VSV-N RNA (left) and HSP-1-V16 RNA (right) were determined by qPCR at 12 hours and 24 hours after infection. **(F)** Viral titres assay of VSV or HSV-1 were determined at 12 hours and 24 hours after infection. Data shown are the mean ±SD. *P < 0.05, **P < 0.01 and ***P < 0.001. Ns, no significant. Data are representative of three independent experiments with similar results.
(TIF)

S5 Fig Overexpression of USP12 in THP-1-Mφs promoted the anti-virus response. THP-1-Mφs transfected with transfected with control or expression vector for USP12 were infected with VSV and HSV virus for 24 hours. Production of IFN-β, CCL5 and IL-6 was determined by ELISA after VSV infection **(A)** or HSV-1 infection **(B)**. QPCR was performed to analyze the expression of ISG15 and MX1 after VSV **(C)** and HSV **(D)** infection. **(E)** Viral VSV-N RNA (left) and HSP-1-V16 RNA (right) were determined by qPCR at 12 hours and 24 hours after infection. **(F)** Viral titres assay of VSV or HSV-1 were determined at 12 hours and 24 hours after infection. Data shown are the mean ±SD. *P < 0.05, **P < 0.01 and ***P < 0.001. Ns, no significant. Data are representative of three independent experiments with similar results.
(TIF)

S6 Fig Related to Fig 3. USP12-deficient mice were susceptible to VSV infection. WT and *Usp12*^-/-^ mice were infected with VSV virus (1 × 10^7^ PFU per mouse). **(A)** Survival rate of WT and *Usp12*^-/-^ mice after VSV infection for indicated time. **(B)** Serum levels of IFN-β and IL-6 was determined by ELISA at 3 days post infection. **(C-E)** Spleen and lung tissues were collected at 3 days post infection. **(C)** Viral VSV-N RNA in spleen and lung tissues were determined by qPCR. **(D)** Expression of IFN-β and IL-6 was determined by qPCR. **(E)** Expression of ISG15, MX1 and IFIT1 was determined by qPCR. Data shown are the mean ±SD. *P < 0.05 and **P < 0.01. Ns, no significant. Data are representative of three independent experiments with similar results.
(TIF)

S7 Fig Related to Fig 4. USP12 deficiency decreased phosphorylation of IRF3 and p65 after HSV infection. WT and *Usp12*^-/-^ BMDMs were infected with HSV-1 **(A)** or VSV **(B)** at indicated times follow by western blot analysis with antibodies against the indicated proteins. Densitometry quantification of band intensity are presented in the right panel. Data shown are the mean ±SD. *P < 0.05, and **P < 0.01. Ns, no significant. Data are representative of three independent experiments with similar results.
(TIF)

S8 Fig Related to Fig 5. USP12 deubiquitinated and stabilized IFI16. **(A)** WT BMDMs were infected HSV-1 for 6 hours followed by immunoprecipitation (IP) using anti-UAF1 or IgG, and immunoblotting (IB) analysis. **(B-C)** WT and *Usp12*^-/-^ BMDMs were infected with CMV for the indicated times. IFI16 expression levels were detected by qPCR **(B)** or western blot **(C)**. Densitometry quantification of band intensity are presented in the right panel. **(D-E)** WT and *Usp12*^-/-^ BMDMs were stimulated with poly(dA:dT) for the indicated times. IFI16 expression levels were detected by qPCR **(D)** or western blot **(E)**. Densitometry quantification of band intensity are presented in the right panel. **(F)** *Uaf1*^*fl/fl*^ and *Uaf1*^*fl/fl*^;Lyz2-Cre BMDMs were infected with HSV-1 for the indicated times. IFI16 expression levels were detected by western blot. Densitometry quantification of band intensity are presented in the right panel. **(G-H)** WT and *Usp12*^-/-^BMDMs were pretreated with STAT1 inhibitor Fludarabine (STAT1 i), and infected with HSV-1 for indicated time. IFI16 expression levels were detected by qPCR **(G)** or western blot **(H)**. Densitometry quantification of band intensity are presented in the below panel. **(I)** IFI16 IB and K48 and K63 ubiquitination analysis using whole-cell extracts of WT and *Usp12*^-/-^ BMDMs infected with HSV-1 for the indicated time. Data shown are the mean ±SD. *P < 0.05, and **P < 0.01. Ns, no significant. Data are representative of three independent experiments with similar results.
(TIF)

S9 Fig Related to Fig 6. USP12 promoted antiviral response dependent on deubiquitive activity in THP-1-Mφ. THP1-Mφ cells were transfected with control or expression vector for V5-USP12 or V5-USP12^C48A^, and infected with HSV-1 for 24 hours. **(A)** Production of IFN-β, CCL5 and IL-6 was determined by ELISA. **(B)** Expression of ISG15, MX1 and IFIT1 was determined by qPCR. **(E)** Viral HSP-1-V16 RNAs were determined by qPCR. (D) Viral titres were determined. Data shown are the mean ±SD. *P < 0.05, **P < 0.01 and ***P < 0.001. Ns, no significant. Data are representative of three independent experiments with similar results.
(TIF)

S10 Fig Related to Fig 7. USP12 regulated ISG responses independent of IFI16. **(A)** WT, *Usp12*^-/-^, *Ifi16*^-/-^ and *Usp12*^-/-^*Ifi16*^-/-^ BMDMs were infected with HSV-1 for 24 hours, and expression of ISG15 and MX1 was determined by qPCR. **(B)** WT, *Usp12*^-/-^, *Ifi16*^-/-^ and *Usp12*^-/-^*Ifi16*^-/-^ BMDMs were stimulated with IFN-β for 6 hours. Expression of ISG15 and MX1 was determined by qPCR. **(C)** WT or *Usp12*^-/-^ BMDMs were transfected with control or expression vector for IFI16, and infected with HSV-1 for 24 hours. Expression of ISG15 and MX1 was determined by qPCR. Data shown are the mean ±SD. **P < 0.01 and ***P < 0.001 by an unpaired *t*-test. Ns, no significant. Data are representative of three independent experiments with similar results.
(TIF)

S11 Fig Illustration of a model on USP12-mediated regulation of innate antiviral signaling. DNA virus infection triggers IFI16-STING signaling which activates IRF3 and p65, and subsequent induction of type I IFNs, chemokines and proinflammatory cytokines. Constant infection also induces ubiquitination and degradation of IFI16. USP12 interacts with IFI16 and inhibited the proteasome- dependent degradation of IFI16 dependent on its deubiquitination activity, thus fine-tune the antiviral response at a certain level to restrict viral infection.
(TIF)

S1 Text Table A. Gene-specific primers used for qRT-PCR. Table B. Antibodies.
(DOCX)
