# Correction: USP12 promotes antiviral responses by deubiquitinating and stabilizing IFI16

**DOI:** 10.1371/journal.ppat.1011827

**Published:** 2023-12-01

**Authors:** Yuling Fu, Xiaoxia Zhan, Xiaolong You, Dingnai Nie, Haiyan Mai, Yitian Chen, Shitong He, Junli Sheng, Zhijie Zeng, Hongwei Li, Jinlong Li, Shengfeng Hu

After this article [1] was published, questions were raised about errors in the gene nomenclature used and missing details in the Methods.

In response to queries about the mouse models, the corresponding author stated that p204/Ifi204 is the mouse ortholog of human IFI16, and that the gene IFI16 was referred to in error, instead of the gene p204. The corresponding author therefore provided the below updated versions of Figs 1–3, S8 Fig, and S10 Fig, where IFI16 has been replaced with p204 in the captions and axes labels. There are also areas throughout the text where IFI16 should be replaced with p204.

The corresponding author stated that the antibody used to detect p204 is NBP2-27153 from Novus and has provided an updated S1 Text file (S3 File) with qPCR primers and Accession ID for p204 (IFI204) here.

The corresponding author provided some clarifying information on the origin of the mouse source: the USP12-deficient (Usp12-/-) mice were generated by Cyagen Biosciences Inc. [2]. P204-/- (or Ifi204-/-) mice were built by Shanghai Research Center for Model Organisms [3]. The corresponding author also provided western blots to support that the ifi204 -/- mice are lacking ifi204 and the double KO mice are missing USP12 and ifi204 in S4 File.

The original underlying data to support all results in the article and Supporting Information files are provided here as S1–S2 Files.

**Fig 5:**
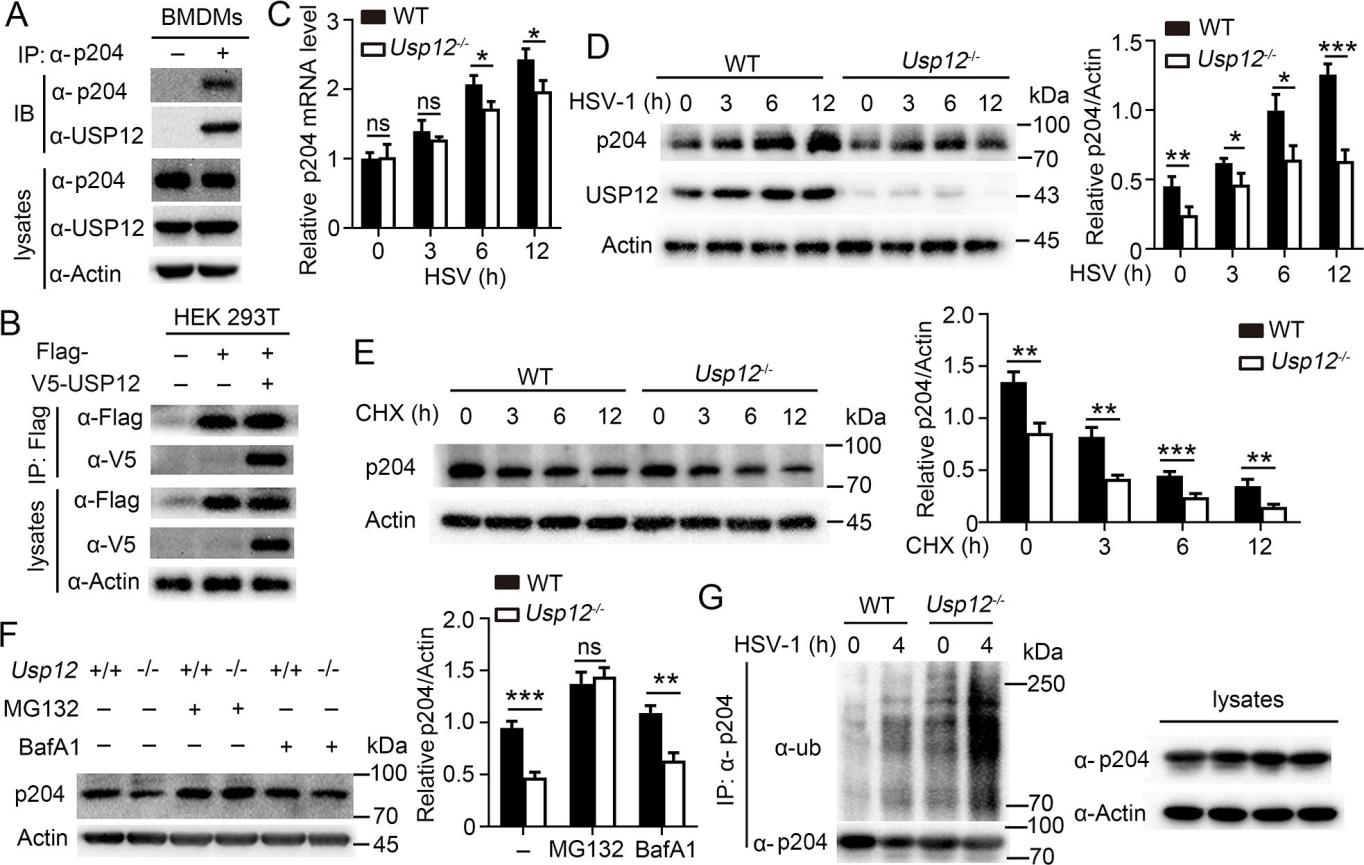
USP12 deubiquitinated and stabilized p204. **(A)** WT BMDMs were infected HSV-1 for 6 hours followed by immunoprecipitation (IP) using anti-p204 or IgG, and immunoblotting (IB) analysis. **(B)** IP and IB analysis of HEK293T cells that were transfected with plasmids encoding V5-USP12 and Flag-p204 for 24 hours. **(C**-**D)** WT and *Usp12*^-/-^ BMDMs were infected with HSV-1 for the indicated times. p204 expression levels were detected by qPCR **(C)** or western blot **(D)**. Densitometry quantification of band intensity are presented in the right panel. **(E)** WT and *Usp12*^-/-^ BMDMs were treated with cycloheximide (CHX, 100 μg/ml) for the indicated times before being analyzed by western blot with antibodies against p204. Densitometry quantification of band intensity are presented in the right panel. **(F)** WT and *Usp12*^-/-^ BMDMs were pretreated with MG132 or BafA1 for 4 hours, infected with HSV-1 for 6 hours, and harvested for subsequent western blot. Densitometry quantification of band intensity are presented in the right panel. **(G)** p204 IB and ubiquitination analysis using whole-cell extracts of WT and *Usp12*^-/-^ BMDMs infected with HSV-1 for the indicated time. Data shown are the mean ±SD. *P < 0.05, **P < 0.01 and ***P < 0.001. Ns, no significant. Data are representative of three independent experiments with similar results.

**Fig 6:**
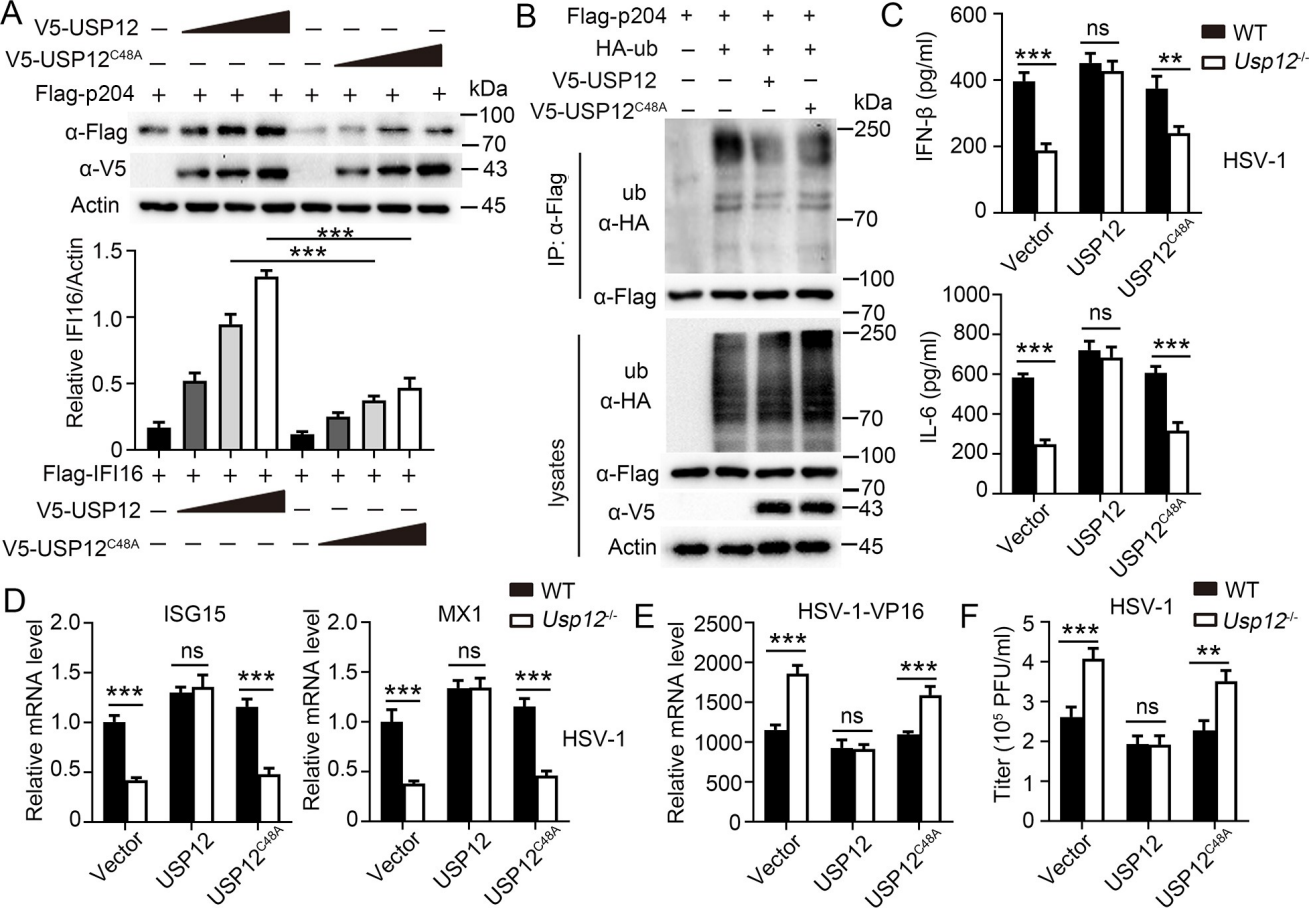
USP12 stabilized p204 dependent on its deubiquitive activity. **(A)** Western blot analysis of extracts of HEK293T cells transfected with p204-Flag vector and increasing doses of expression vector for V5-USP12 or V5-USP12^C48A^. Densitometry quantification of band intensity are presented in the below panel. **(B)** Immunoblot (IB) and ubiquitination analysis of extracts of HEK293T cells transfected with indicated plasmids. **(C**-**E)** WT or *Usp12*^-/-^ BMDMs were transfected with control or expression vector for V5-USP12 or V5-USP12^C48A^, and infected with HSV-1for 24h. **(C)** Production of IFN-β and IL-6 was determined by ELISA. **(D)** Expression of ISG15 and MX1 was determined by qPCR. **(E)** Viral HSP-1-V16 RNAs were determined by qPCR. **(F)** Viral titres were determined. Data shown are the mean ±SD. *P < 0.05, **P < 0.01 and ***P < 0.001. Ns, no significant. Data are representative of three independent experiments with similar results.

**Fig 7:**
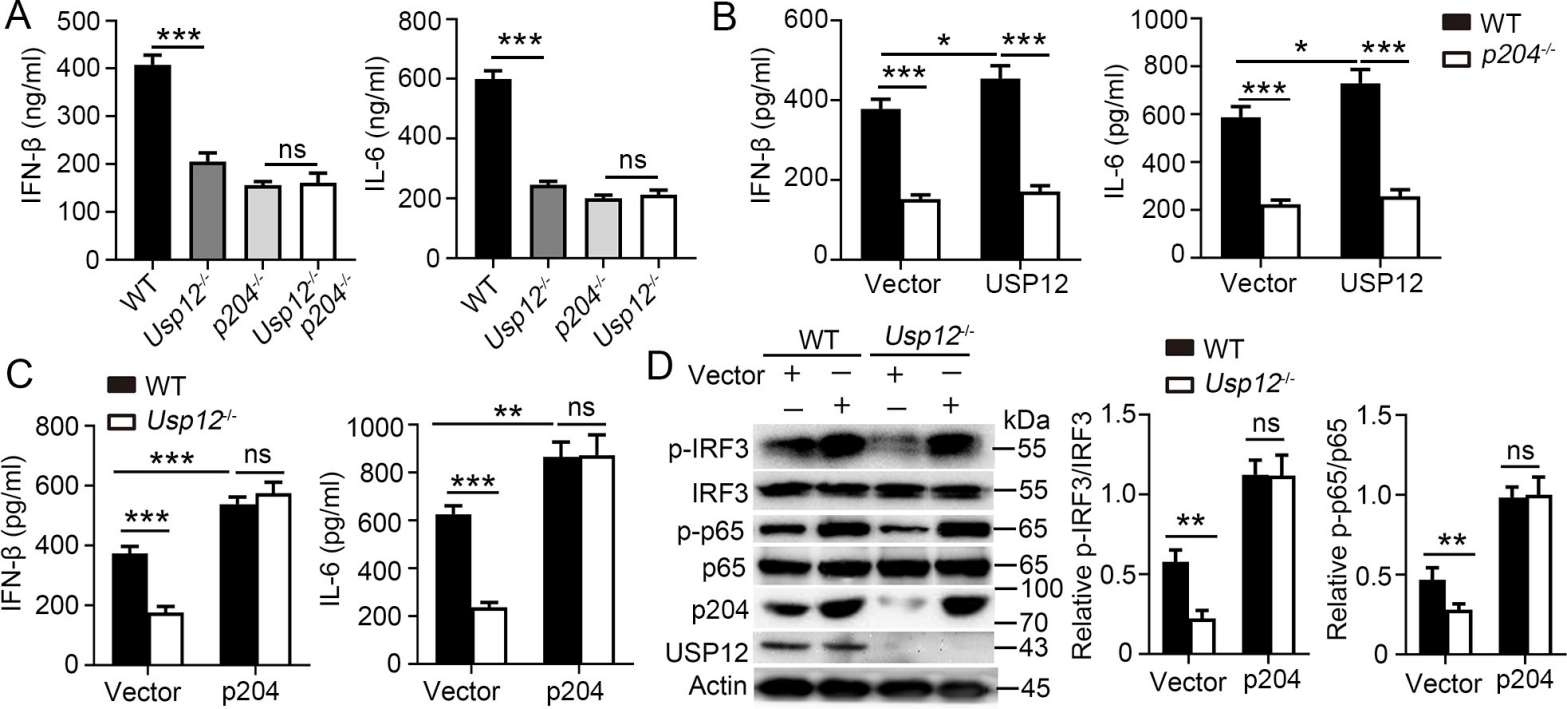
USP12 function dependent on p204. **(A)** ELISA analysis of IFN-β and IL-6 in the supernatants of WT, *Usp12*^-/-^, p204^-/-^ and *Usp12*^-/-^p204^-/-^ BMDMs infected with HSV-1 for 24 h. **(B)** ELISA analysis of IFN-β and IL-6 in the supernatants of WT or p204^-/-^ BMDMs transfected with control, or expression vector for USP12. **(C)** ELISA analysis of IFN-β and IL-6 in the supernatants of WT or *Usp12*^-/-^ BMDMs transfected with control or expression vector for p204. **(D)** Western blot analysis of extracts of WT or *Usp*12^-/-^ BMDMs transfected with indicated vectors. Densitometry quantification of band intensity are presented in the right panel. Data shown are the mean ±SD. *P < 0.05, **P < 0.01 and ***P < 0.001. Ns, no significant. Data are representative of three independent experiments with similar results.

## Supporting information

S1 File Underlying data to support results in article [1]. (XLSX)

S2 File Underlying image data to support the western blots in Figs 1–3, S1 Fig, S3 Fig, and S7-S8 Figs. (DOCX)

S3 File Table A. Gene-specific primers used for qRT-PCR. Table B. Antibodies.
(DOCX)

S4 File Western blot showing gene knockout as described in article [1]. (TIF)

S1 Fig Related to Fig 5. USP12 deubiquitinated and stabilized p204. **(A)** WT BMDMs were infected HSV-1 for 6 hours followed by immunoprecipitation (IP) using anti-UAF1 or IgG, and immunoblotting (IB) analysis. **(B**-**C)** WT and *Usp12*^-/-^ BMDMs were infected with CMV for the indicated times. p204 expression levels were detected by qPCR **(B)** or western blot **(C)**. Densitometry quantification of band intensity are presented in the right panel. **(D**-**E)** WT and *Usp12*^-/-^ BMDMs were stimulated with poly(dA:dT) for the indicated times. p204 expression levels were detected by qPCR **(D)** or western blot **(E)**. Densitometry quantification of band intensity are presented in the right panel. **(F)** *Uaf1*^*fl/fl*^ and *Uaf1*^*fl/fl*^;Lyz2-Cre BMDMs were infected with HSV-1 for the indicated times. p204 expression levels were detected by western blot. Densitometry quantification of band intensity are presented in the right panel. **(G**-**H)** WT and *Usp12*^-/-^BMDMs were pretreated with STAT1 inhibitor Fludarabine (STAT1 i), and infected with HSV-1 for indicated time. p204 expression levels were detected by qPCR **(G)** or western blot **(H)**. Densitometry quantification of band intensity are presented in the below panel. **(I)** p204 IB and K48 and K63 ubiquitination analysis using whole-cell extracts of WT and *Usp12*^-/-^ BMDMs infected with HSV-1 for the indicated time. Data shown are the mean ±SD. *P < 0.05, and **P < 0.01. Ns, no significant. Data are representative of three independent experiments with similar results.
(TIF)

S2 Fig Related to Fig 7. USP12 regulated ISG responses independent of p204. **(A)** WT, *Usp12*^-/-^, *p204*^-/-^ and *Usp12*^-/-^*p204*^-/-^ BMDMs were infected with HSV-1 for 24 hours, and expression of ISG15 and MX1 was determined by qPCR. **(B)** WT, *Usp12*^-/-^, *p204*^-/-^ and *Usp12*^-/-^*p204*^-/-^ BMDMs were stimulated with IFN-β for 6 hours. Expression of ISG15 and MX1 was determined by qPCR. **(C)** WT or *Usp12*^-/-^ BMDMs were transfected with control or expression vector for p204, and infected with HSV-1 for 24 hours. Expression of ISG15 and MX1 was determined by qPCR. Data shown are the mean ±SD. **P < 0.01 and ***P < 0.001 by an unpaired *t*-test. Ns, no significant. Data are representative of three independent experiments with similar results.
(TIF)
