# Supplementary figures and images for: USP12 promotes antiviral responses by deubiquitinating and stabilizing IFI16
Source: PLoS Pathog. 2023 Jul 6;19(7):e1011480. doi: 10.1371/journal.ppat.1011480 (PMC10353808; doi:10.1371/journal.ppat.1011480)

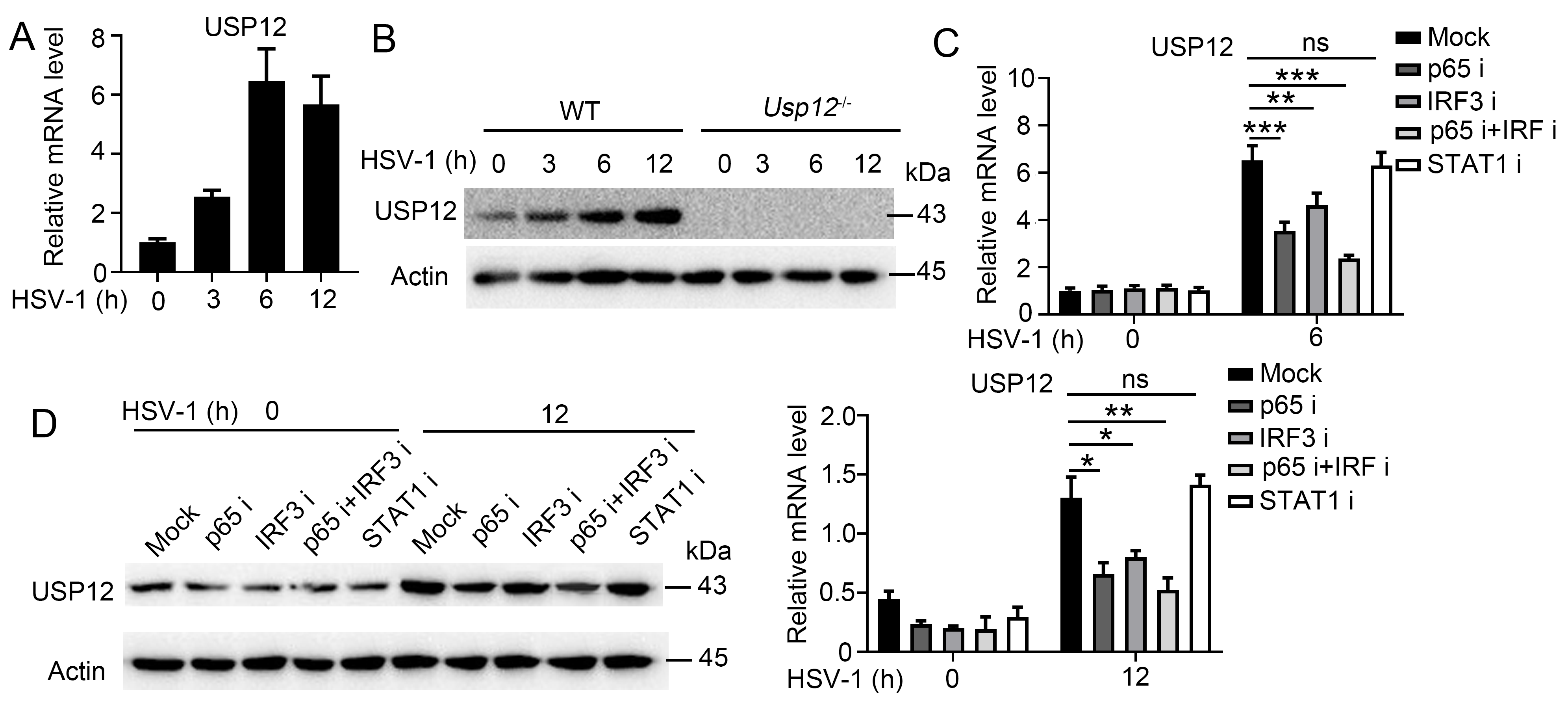

Supplement: S1 Fig — (A) The mRNA expression of USP12 were assessed using qPCR analysis in WT mouse BMDMs infected with HSV-1 for indicated time. (B) The protein abundance of USP12 were assessed using western blot in BMDMs infected with infected with HSV-1 for indicated time. (C-D) WT BMDMs were pretreated with NF-κB p65 inhibitor JSH-23 (p65 i), IRF3 inhibitor Geldanamycin (IRF3 i), or STAT1 inhibitor Fludarabine (STAT1 i), and infected with HSV-1 for indicated time. (C) The mRNA expression of USP12 were assessed using qPCR. (D) The protein abundance of USP12 were assessed using western blot. Data shown are the mean ±SD. *P < 0.05, **P < 0.01 and ***P < 0.001. Ns, no significant. Data are representative of three independent experiments with similar results. (TIF) [file ppat.1011480.s001.tif]

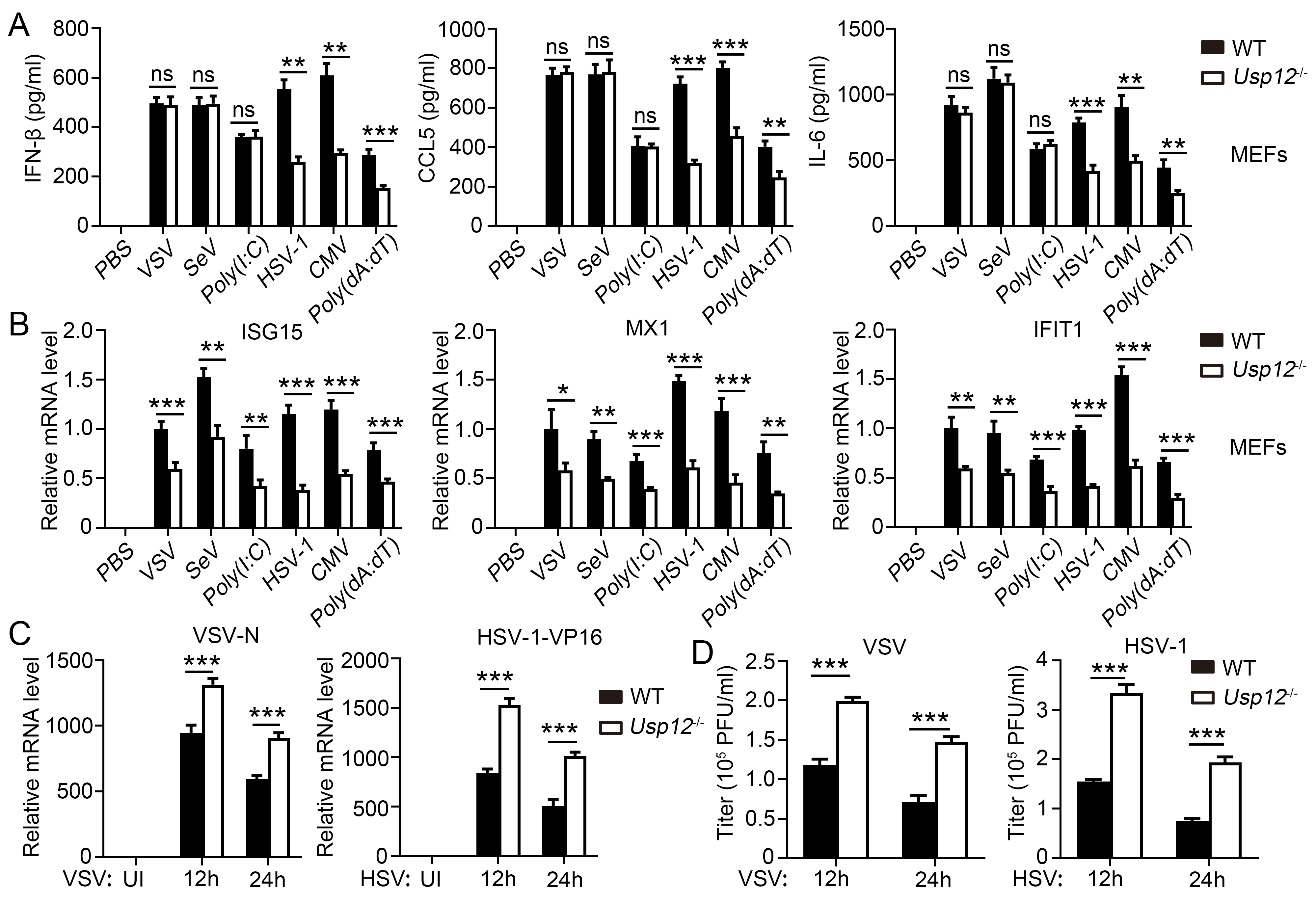

Supplement: S2 Fig — WT or Usp12-/- MEFs were infected with VSV, SEV, HSV-1 or CMV, or transfected with Poly (I:C) or Poly(dA:dT) for 24 house or the indicated time. (A) ELISA analysis of IFN-β, CCL5 and IL-6 in the supernatants. (B) The expression of ISG15, MX1 and IFIT1 was analyzed by qPCR. (C) Viral VSV-N RNA (left) and HSP-1-V16 RNA (right) were determined by qPCR. (D) Viral titres of VSV or HSV virus were determined. Data shown are the mean ±SD. *P < 0.05, **P < 0.01 and ***P < 0.001. Ns, no significant. Data are representative of three independent experiments with similar results. (TIF) [file ppat.1011480.s002.tif]

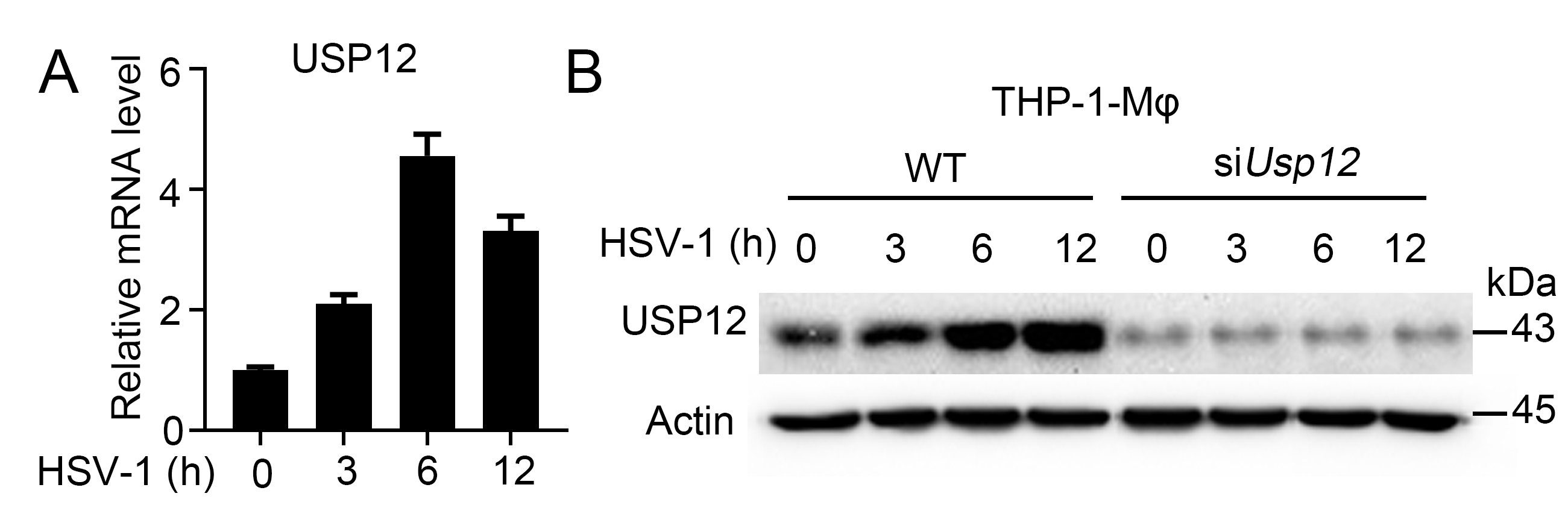

Supplement: S3 Fig — (A) The mRNA expression of USP12 were assessed using qPCR analysis in THP-1-Mφs infected with HSV-1 for indicated time. (B) The protein abundance of USP12 were assessed using western blot in THP-1-Mφs infected with infected with HSV-1 for indicated time. Data shown are the mean ±SD. Data are representative of three independent experiments with similar results. (TIF) [file ppat.1011480.s003.tif]

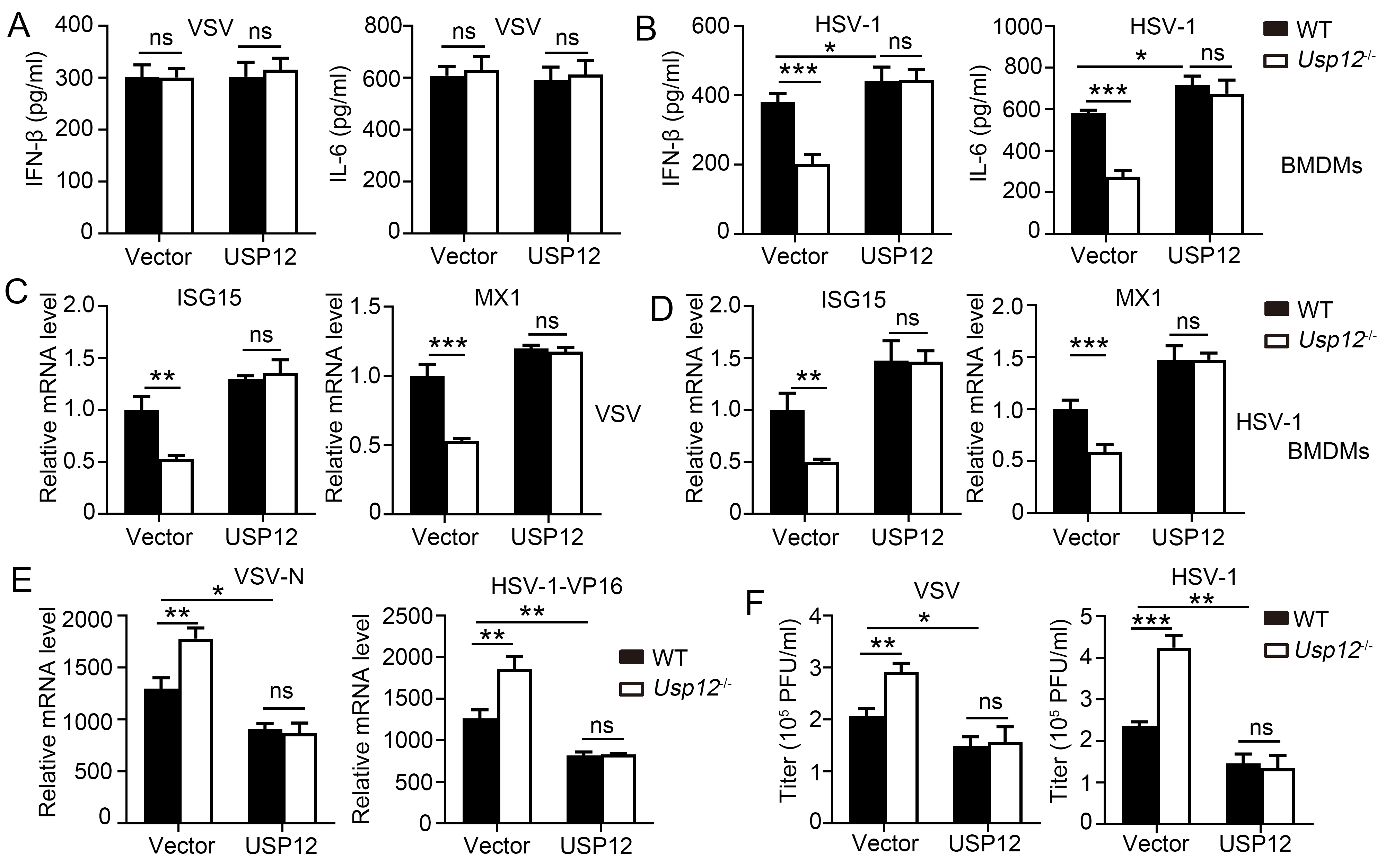

Supplement: S4 Fig — WT or Usp12-/- BMDMs transfected with control or expression vector for USP12 were infected with VSV or HSV virus for 24 hours. Production of IFN-β and IL-6 was determined by ELISA after VSV infection (A) or HSV-1 infection (B). QPCR was performed to analyze the expression of ISG15 and MX1 after VSV (C) and HSV (D) infection. (E) Viral VSV-N RNA (left) and HSP-1-V16 RNA (right) were determined by qPCR at 12 hours and 24 hours after infection. (F) Viral titres assay of VSV or HSV-1 were determined at 12 hours and 24 hours after infection. Data shown are the mean ±SD. *P < 0.05, **P < 0.01 and ***P < 0.001. Ns, no significant. Data are representative of three independent experiments with similar results. (TIF) [file ppat.1011480.s004.tif]

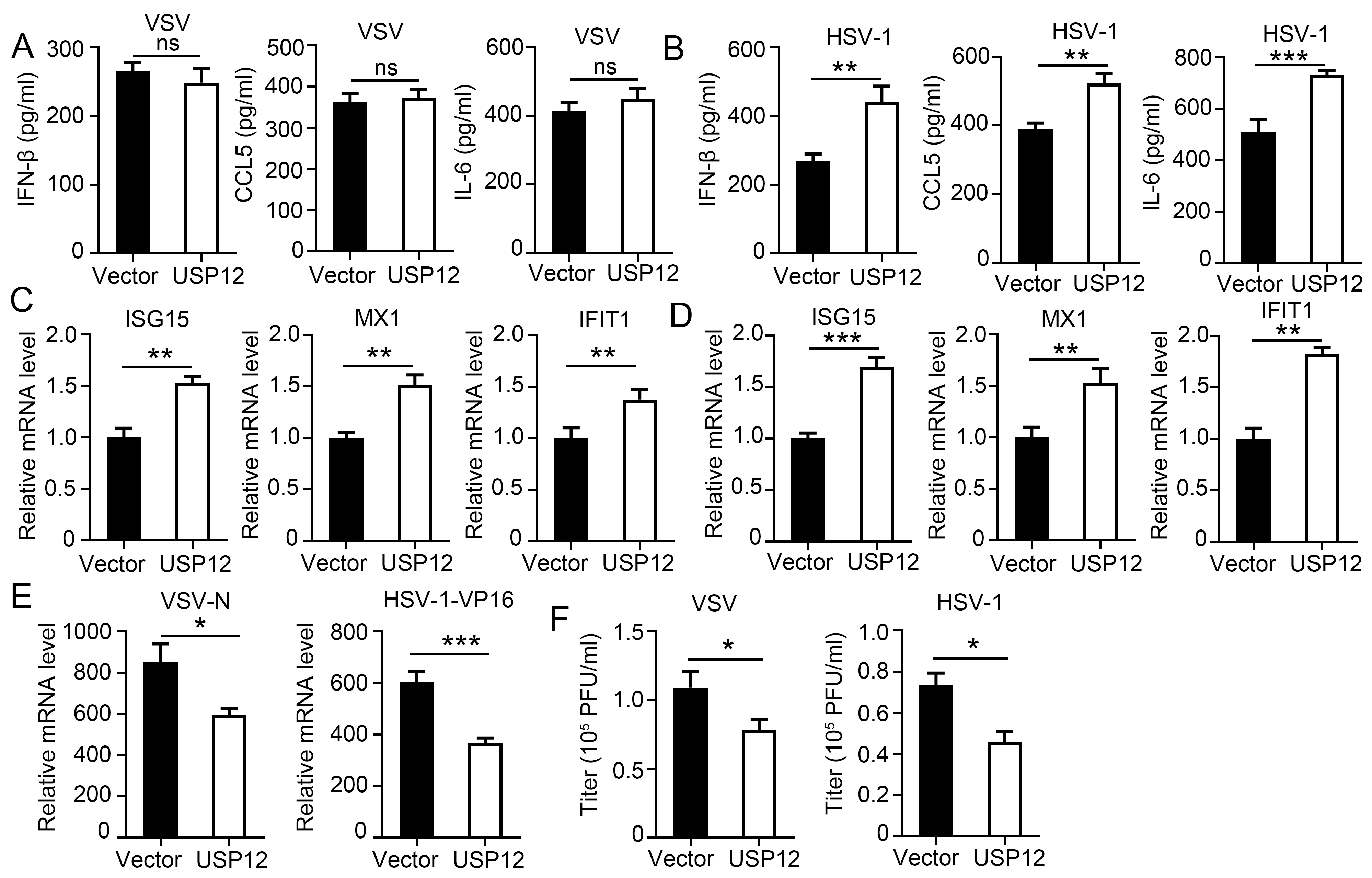

Supplement: S5 Fig — THP-1-Mφs transfected with transfected with control or expression vector for USP12 were infected with VSV and HSV virus for 24 hours. Production of IFN-β, CCL5 and IL-6 was determined by ELISA after VSV infection (A) or HSV-1 infection (B). QPCR was performed to analyze the expression of ISG15 and MX1 after VSV (C) and HSV (D) infection. (E) Viral VSV-N RNA (left) and HSP-1-V16 RNA (right) were determined by qPCR at 12 hours and 24 hours after infection. (F) Viral titres assay of VSV or HSV-1 were determined at 12 hours and 24 hours after infection. Data shown are the mean ±SD. *P < 0.05, **P < 0.01 and ***P < 0.001. Ns, no significant. Data are representative of three independent experiments with similar results. (TIF) [file ppat.1011480.s005.tif]

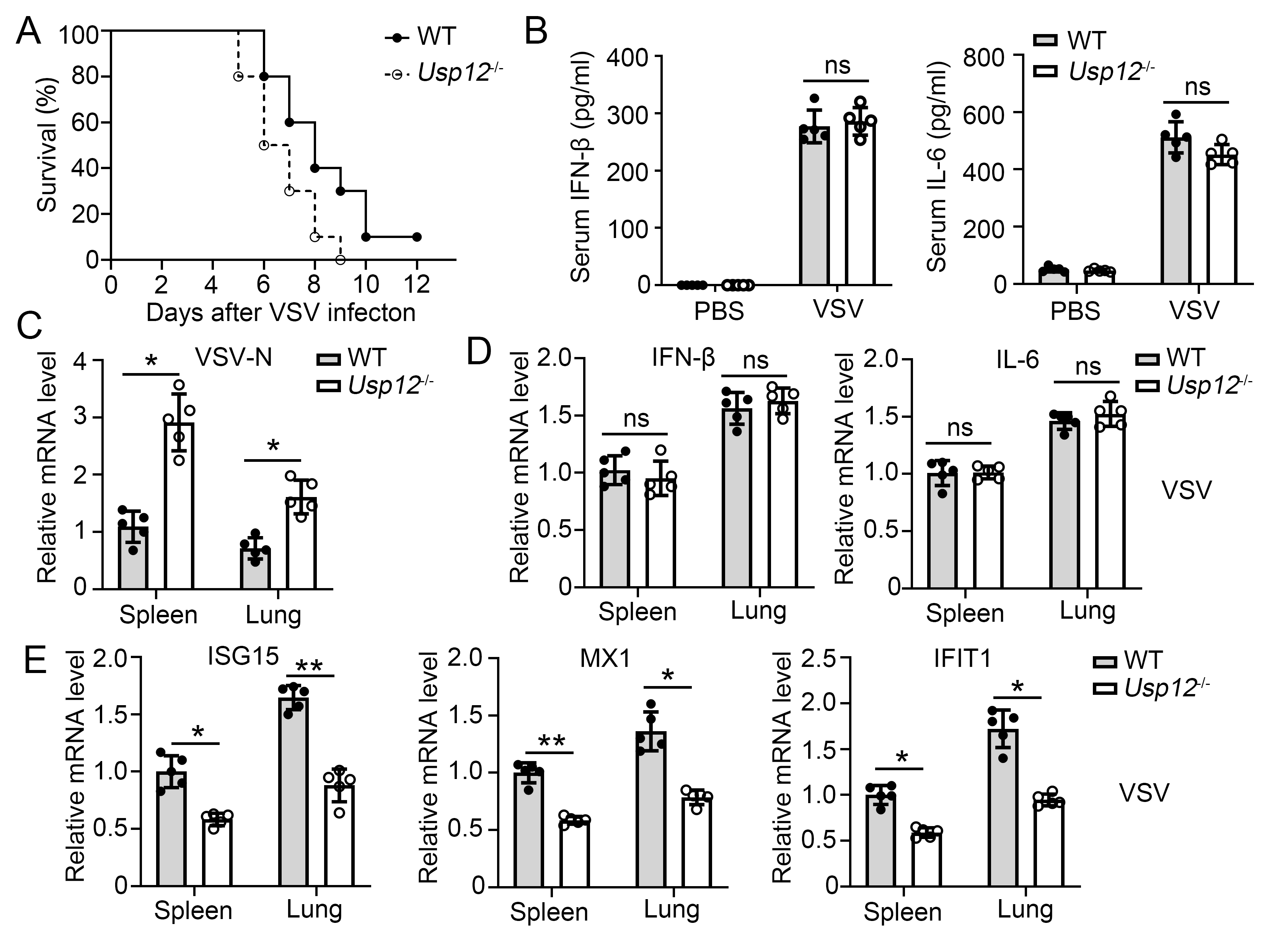

Supplement: S6 Fig — WT and Usp12-/- mice were infected with VSV virus (1 × 10^7^ PFU per mouse). (A) Survival rate of WT and Usp12-/- mice after VSV infection for indicated time. (B) Serum levels of IFN-β and IL-6 was determined by ELISA at 3 days post infection. (C-E) Spleen and lung tissues were collected at 3 days post infection. (C) Viral VSV-N RNA in spleen and lung tissues were determined by qPCR. (D) Expression of IFN-β and IL-6 was determined by qPCR. (E) Expression of ISG15, MX1 and IFIT1 was determined by qPCR. Data shown are the mean ±SD. *P < 0.05 and **P < 0.01. Ns, no significant. Data are representative of three independent experiments with similar results. (TIF) [file ppat.1011480.s006.tif]

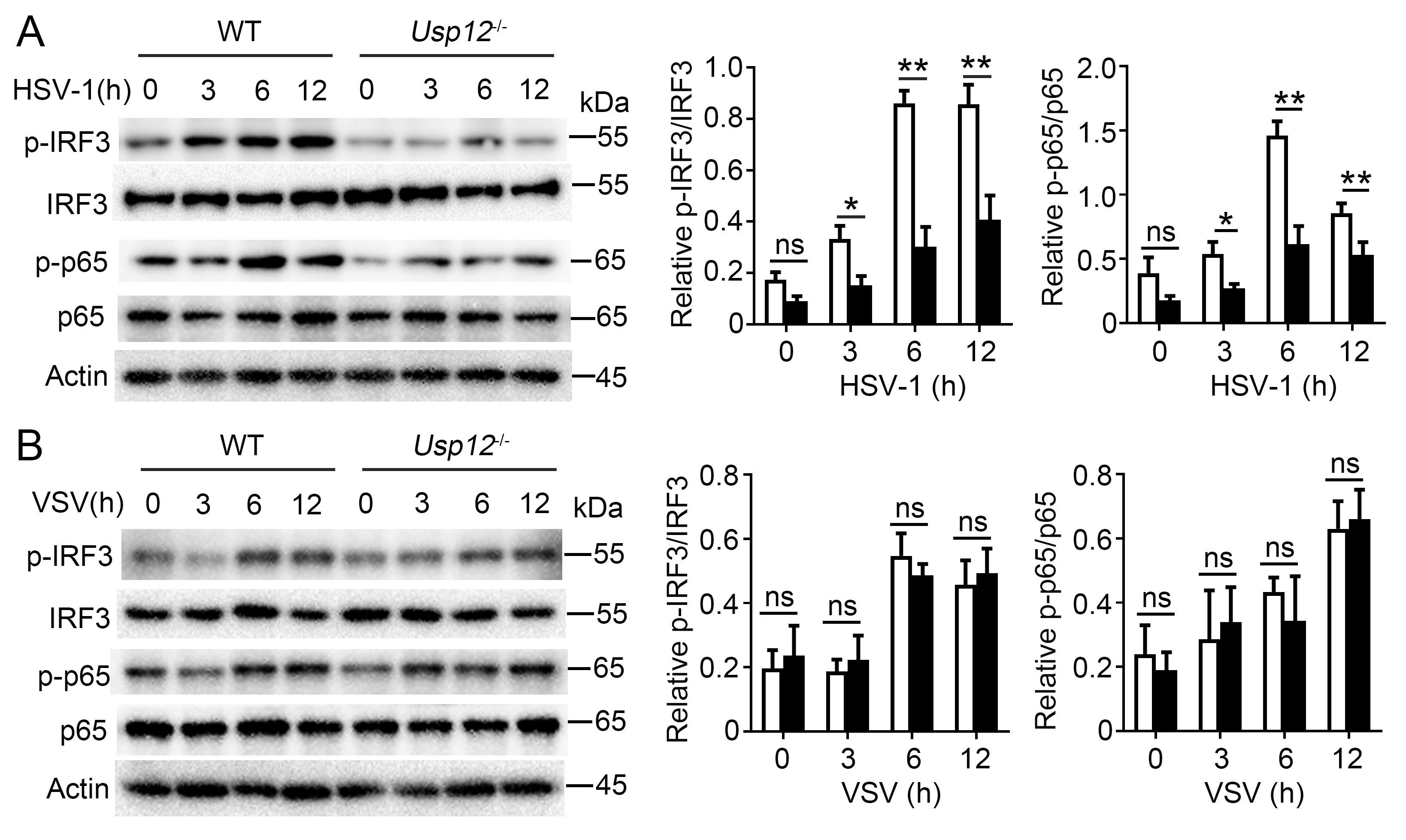

Supplement: S7 Fig — WT and Usp12-/- BMDMs were infected with HSV-1 (A) or VSV (B) at indicated times follow by western blot analysis with antibodies against the indicated proteins. Densitometry quantification of band intensity are presented in the right panel. Data shown are the mean ±SD. *P < 0.05, and **P < 0.01. Ns, no significant. Data are representative of three independent experiments with similar results. (TIF) [file ppat.1011480.s007.tif]

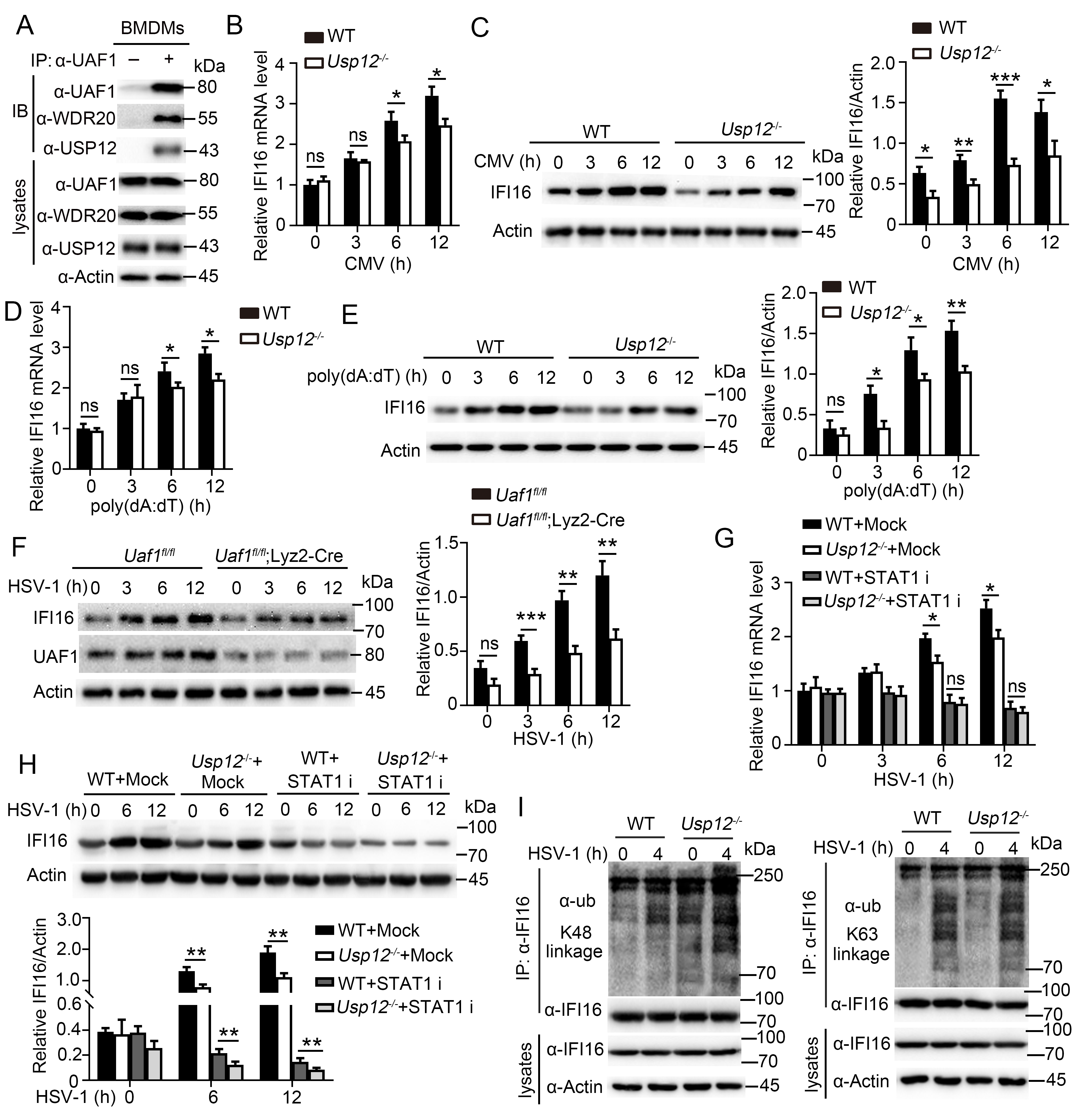

Supplement: S8 Fig — (A) WT BMDMs were infected HSV-1 for 6 hours followed by immunoprecipitation (IP) using anti-UAF1 or IgG, and immunoblotting (IB) analysis. (B-C) WT and Usp12-/- BMDMs were infected with CMV for the indicated times. IFI16 expression levels were detected by qPCR (B) or western blot (C). Densitometry quantification of band intensity are presented in the right panel. (D-E) WT and Usp12-/- BMDMs were stimulated with poly(dA:dT) for the indicated times. IFI16 expression levels were detected by qPCR (D) or western blot (E). Densitometry quantification of band intensity are presented in the right panel. (F) Uaf1fl/fl and Uaf1fl/fl;Lyz2-Cre BMDMs were infected with HSV-1 for the indicated times. IFI16 expression levels were detected by western blot. Densitometry quantification of band intensity are presented in the right panel. (G-H) WT and Usp12-/-BMDMs were pretreated with STAT1 inhibitor Fludarabine (STAT1 i), and infected with HSV-1 for indicated time. IFI16 expression levels were detected by qPCR (G) or western blot (H). Densitometry quantification of band intensity are presented in the below panel. (I) IFI16 IB and K48 and K63 ubiquitination analysis using whole-cell extracts of WT and Usp12-/- BMDMs infected with HSV-1 for the indicated time. Data shown are the mean ±SD. *P < 0.05, and **P < 0.01. Ns, no significant. Data are representative of three independent experiments with similar results. (TIF) [file ppat.1011480.s008.tif]

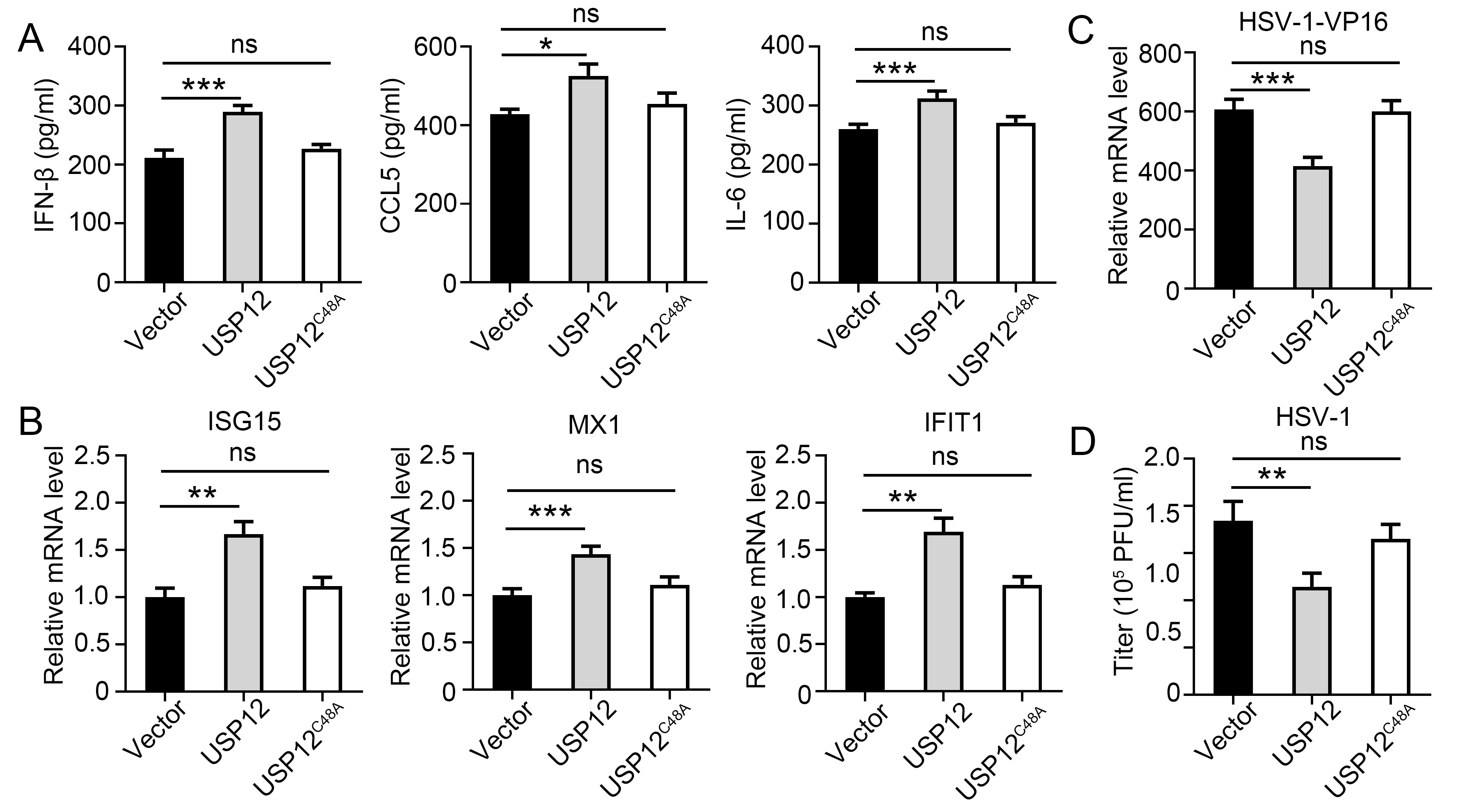

Supplement: S9 Fig — THP1-Mφ cells were transfected with control or expression vector for V5-USP12 or V5-USP12C48A, and infected with HSV-1 for 24 hours. (A) Production of IFN-β, CCL5 and IL-6 was determined by ELISA. (B) Expression of ISG15, MX1 and IFIT1 was determined by qPCR. (E) Viral HSP-1-V16 RNAs were determined by qPCR. (D) Viral titres were determined. Data shown are the mean ±SD. *P < 0.05, **P < 0.01 and ***P < 0.001. Ns, no significant. Data are representative of three independent experiments with similar results. (TIF) [file ppat.1011480.s009.tif]

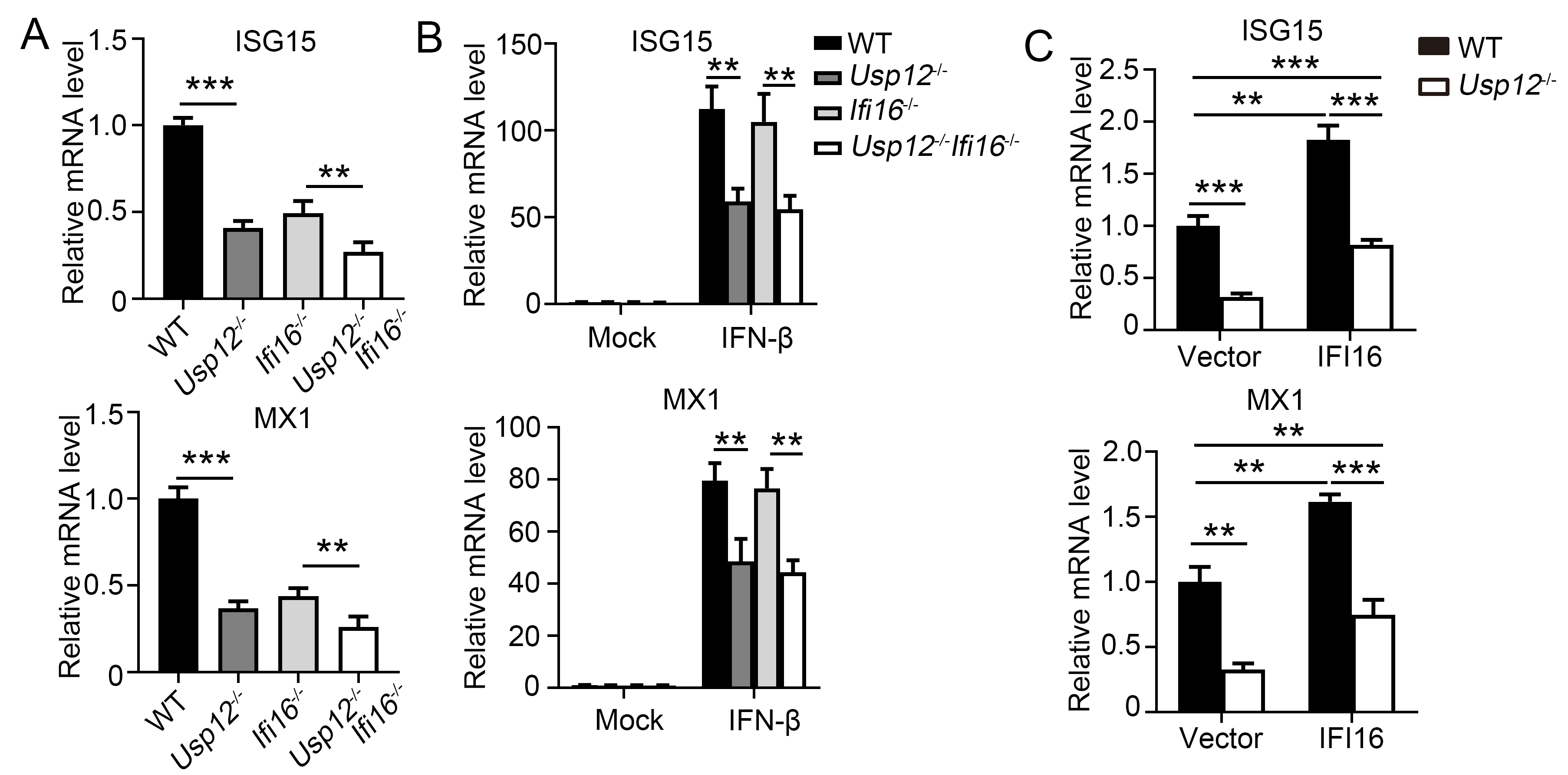

Supplement: S10 Fig — (A) WT, Usp12-/-, Ifi16-/- and Usp12-/-Ifi16-/- BMDMs were infected with HSV-1 for 24 hours, and expression of ISG15 and MX1 was determined by qPCR. (B) WT, Usp12-/-, Ifi16-/- and Usp12-/-Ifi16-/- BMDMs were stimulated with IFN-β for 6 hours. Expression of ISG15 and MX1 was determined by qPCR. (C) WT or Usp12-/- BMDMs were transfected with control or expression vector for IFI16, and infected with HSV-1 for 24 hours. Expression of ISG15 and MX1 was determined by qPCR. Data shown are the mean ±SD. **P < 0.01 and ***P < 0.001 by an unpaired t-test. Ns, no significant. Data are representative of three independent experiments with similar results. (TIF) [file ppat.1011480.s010.tif]

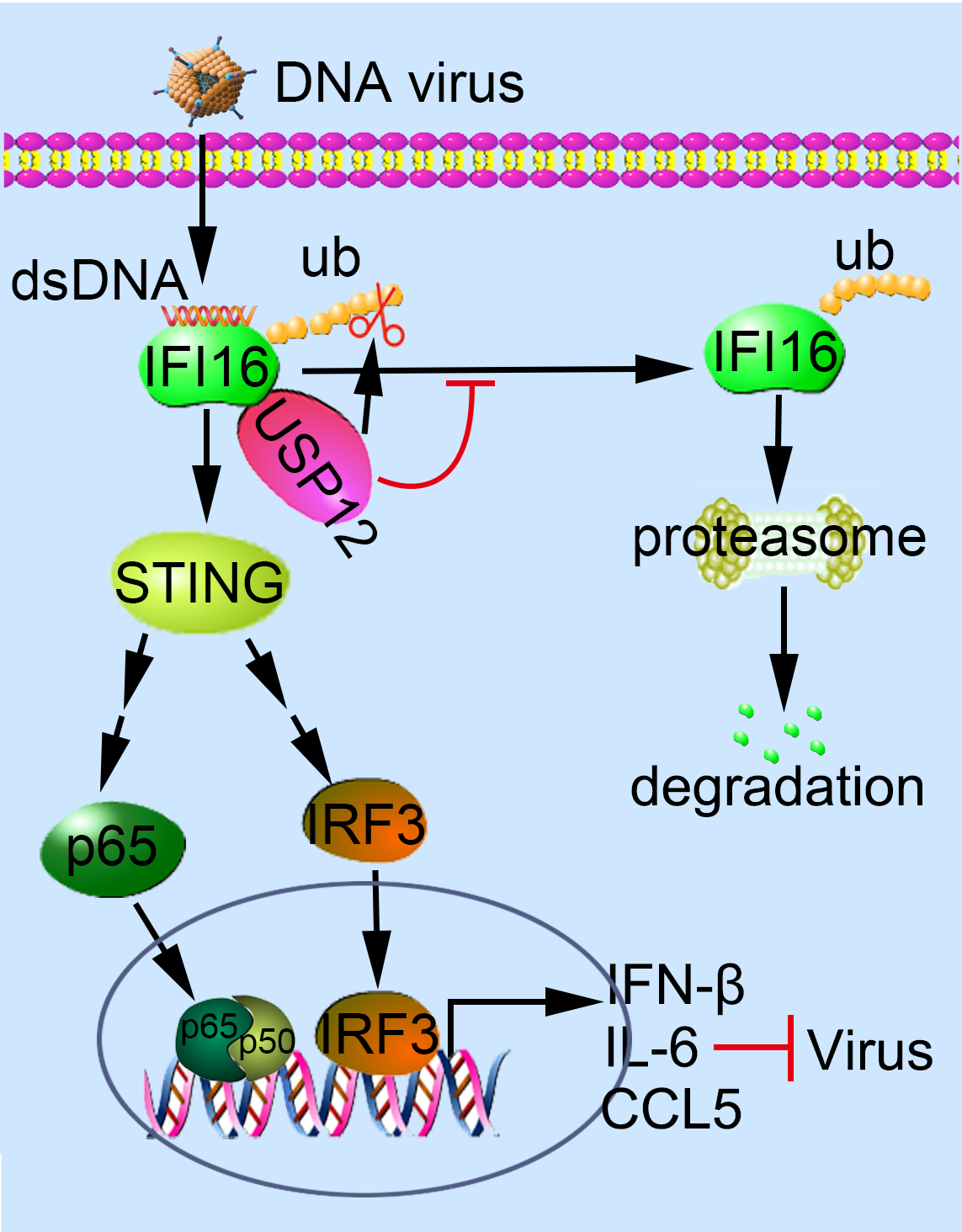

Supplement: S11 Fig — DNA virus infection triggers IFI16-STING signaling which activates IRF3 and p65, and subsequent induction of type I IFNs, chemokines and proinflammatory cytokines. Constant infection also induces ubiquitination and degradation of IFI16. USP12 interacts with IFI16 and inhibited the proteasome- dependent degradation of IFI16 dependent on its deubiquitination activity, thus fine-tune the antiviral response at a certain level to restrict viral infection. (TIF) [file ppat.1011480.s011.tif]
