# Supplementary material for: USP12 promotes antiviral responses by deubiquitinating and stabilizing IFI16
Source: PLoS Pathog. 2023 Jul 6;19(7):e1011480. doi: 10.1371/journal.ppat.1011480 (PMC10353808; doi:10.1371/journal.ppat.1011480)
Supplement: S1 Text — Table A. Gene-specific primers used for qRT-PCR. Table B. Antibodies. (DOCX) [file ppat.1011480.s012.docx]

**Table A. Gene-specific primers used for qRT-PCR**

| **Gene** | **Forward primer** | **Reverse primer** |
| --- | --- | --- |
| *mActb* | CATTGCTGACAGGATGCAGAAGG | TGCTGGAAGGTGGACAGTGAGG |
| *mIfnb* | CTTGGATTCCTACAAAGAAGCAGC | TCCTCCTTCTGGAACTGCTGCA |
| *mIl6* | TACCACTTCACAAGTCGGAGGC | CTGCAAGTGCATCATCGTTGTTC |
| *mIsg15* | CATCCTGGTGAGGAACGAAAGG | CTCAGCCAGAACTGGTCTTCGT |
| *mMx1* | TGGACATTGCTACCACAGAGGC | TTGCCTTCAGCACCTCTGTCCA |
| *mIfit1* | TACAGGCTGGAGTGTGCTGAGA | CTCCACTTTCAGAGCCTTCGCA |
| *hGapdh* | GTCTCCTCTGACTTCAACAGCG | ACCACCCTGTTGCTGTAGCCAA |
| *hIfnb* | CTTGGATTCCTACAAAGAAGCAGC | TCCTCCTTCTGGAACTGCTGCA |
| *hIl6* | AGACAGCCACTCACCTCTTCAG | TTCTGCCAGTGCCTCTTTGCTG |
| *hIsg15* | CTCTGAGCATCCTGGTGAGGAA | AAGGTCAGCCAGAACAGGTCGT |
| *hMx1* | GGCTGTTTACCAGACTCCGACA | CACAAAGCCTGGCAGCTCTCTA |
| *hIfit1* | GCCTTGCTGAAGTGTGGAGGAA | ATCCAGGCGATAGGCAGAGATC |

m, Mouse; h, Human

**Table B. Antibodies**

| **Antigen** | **Reactivity** | **Clone** | **Manufacture** | **Use** |
| --- | --- | --- | --- | --- |
| IFI16 | H | 1G7 | Santa Cruz | WB |
| IFI16 | M | NBP3-10994 | Novus | WB |
| USP12 | H/M | polyclonal | Sigma | WB |
| p-IRF3 | H/M | D6O1M | CST | WB |
| IRF3 | H/M | D614C | CST | WB |
| p-p65 | H/M | 93H1 | CST | WB |
| P65 | H/M | D14E12 | CST | WB |
| Ubiquitin | H/M | P4D1 | CST | WB |
| FLAG | H/M | D6W5B | CST | WB |
| HA | H/M | C29F4 | CST | WB |
| Myc | H/M | 9B11 | CST | WB |
| β-Actin | H/M | D6A8 | CST | WB |

M, Mouse; H, Human
