# Supplementary material for: Correction: USP12 promotes antiviral responses by deubiquitinating and stabilizing IFI16
Source: PLoS Pathog. 2023 Dec 1;19(12):e1011827. doi: 10.1371/journal.ppat.1011827 (PMC10691705; doi:10.1371/journal.ppat.1011827)
Supplement: S2 File — (DOCX) [file ppat.1011827.s002.docx]

**Figure 5A**


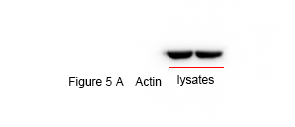

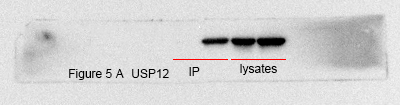

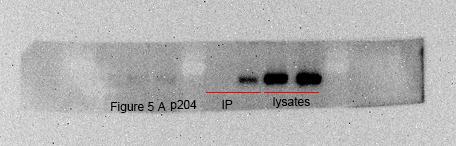


**Figure 5B**


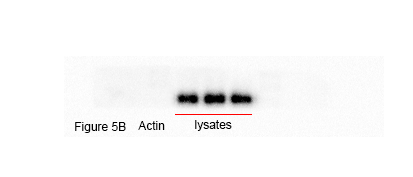

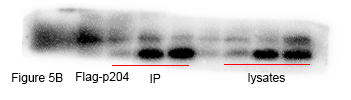

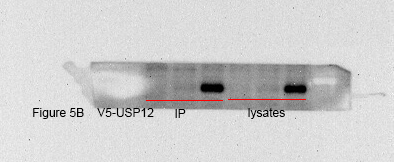


**Figure 5D**


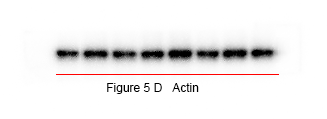

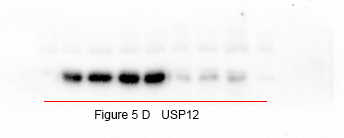

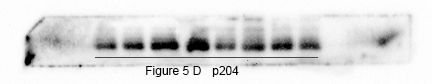


**Figure 5E**


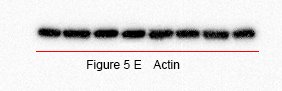

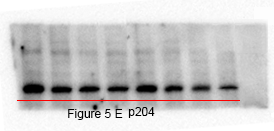


**Figure 5F**


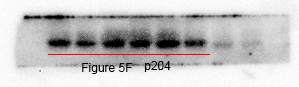

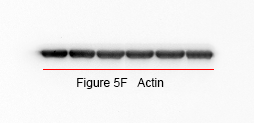


**Figure 5G**


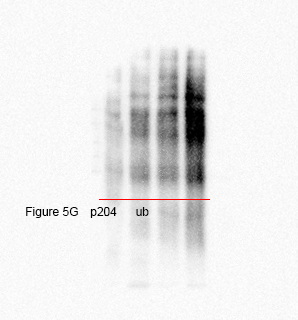

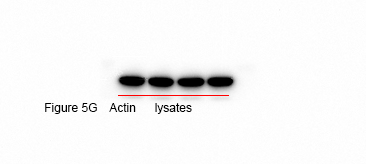

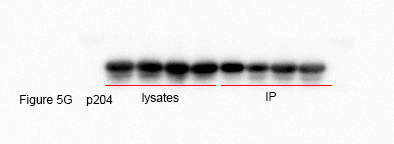


**Figure 6A**


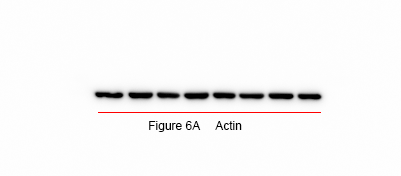

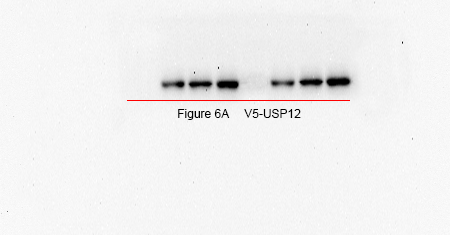

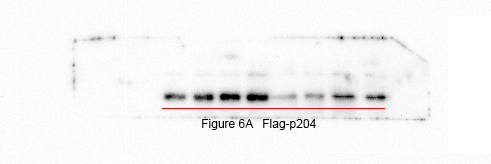


**Figure 6B**


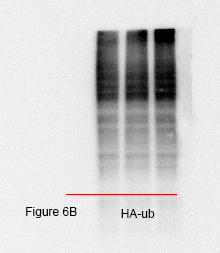

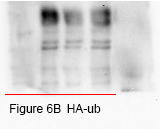


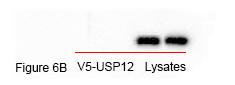

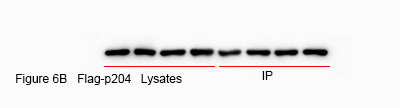


**Figure 7D**


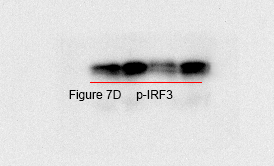

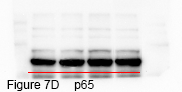

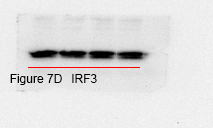

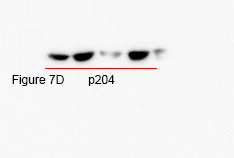

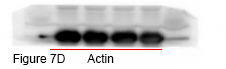

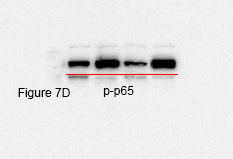


**S1_Fig. B**


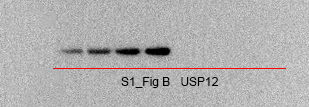

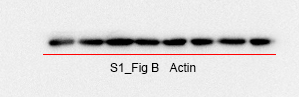


**S1_Fig. D**

**
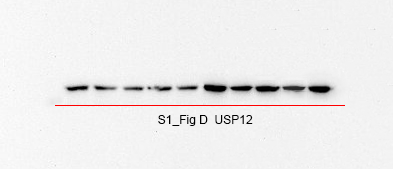

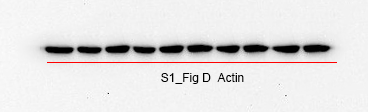
**

**S3_Fig. B**


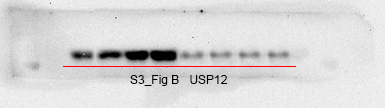

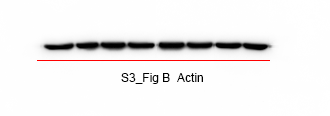


**S7_Fig. A**


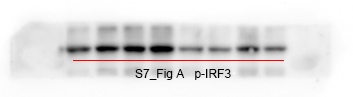

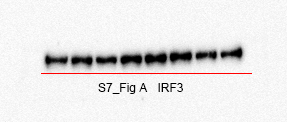

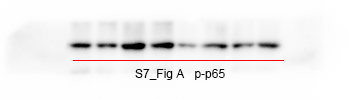

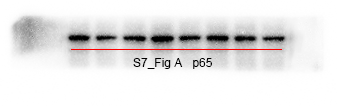

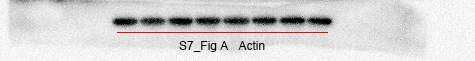


**S7_Fig. C**


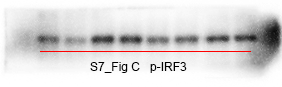

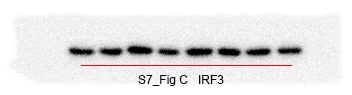

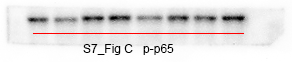

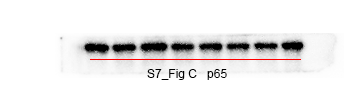

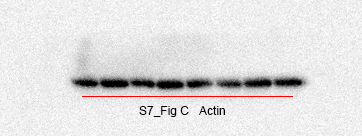


**S8_Fig. A**


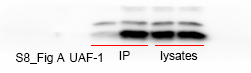

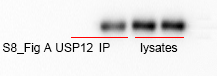

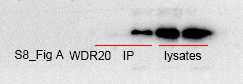

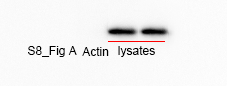


**S8_Fig. C**

**
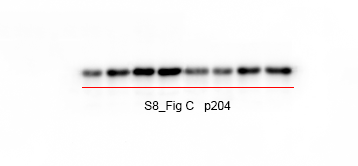

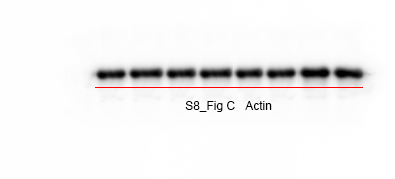
**

**S8_Fig. E**

**
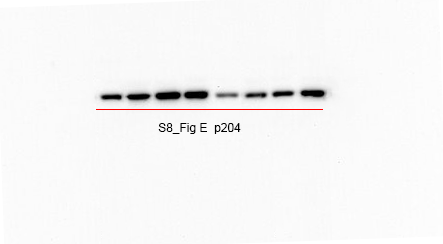

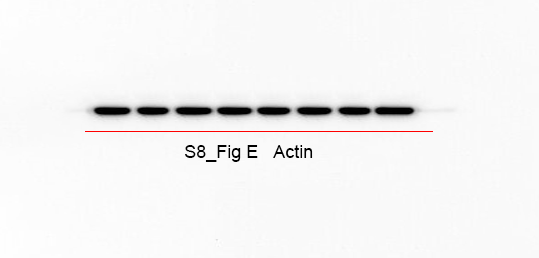
**

**S8_Fig. F**

**
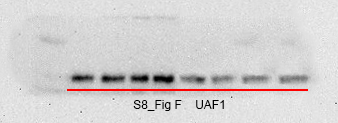

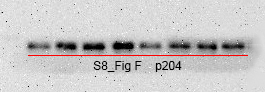

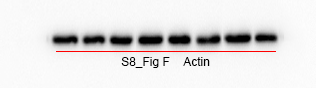
**

**S8_Fig. H**

**
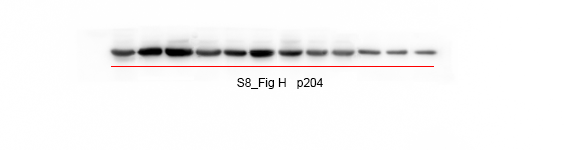

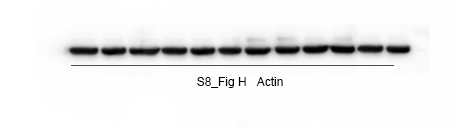
**
