# Supplementary figures and images for: Correction: USP12 promotes antiviral responses by deubiquitinating and stabilizing IFI16
Source: PLoS Pathog. 2023 Dec 1;19(12):e1011827. doi: 10.1371/journal.ppat.1011827 (PMC10691705; doi:10.1371/journal.ppat.1011827)

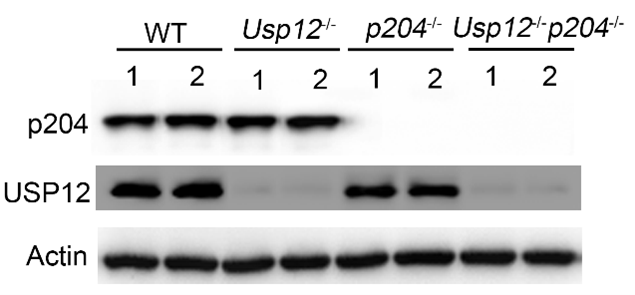

Supplement: S4 File — (TIF) [file ppat.1011827.s004.tif]

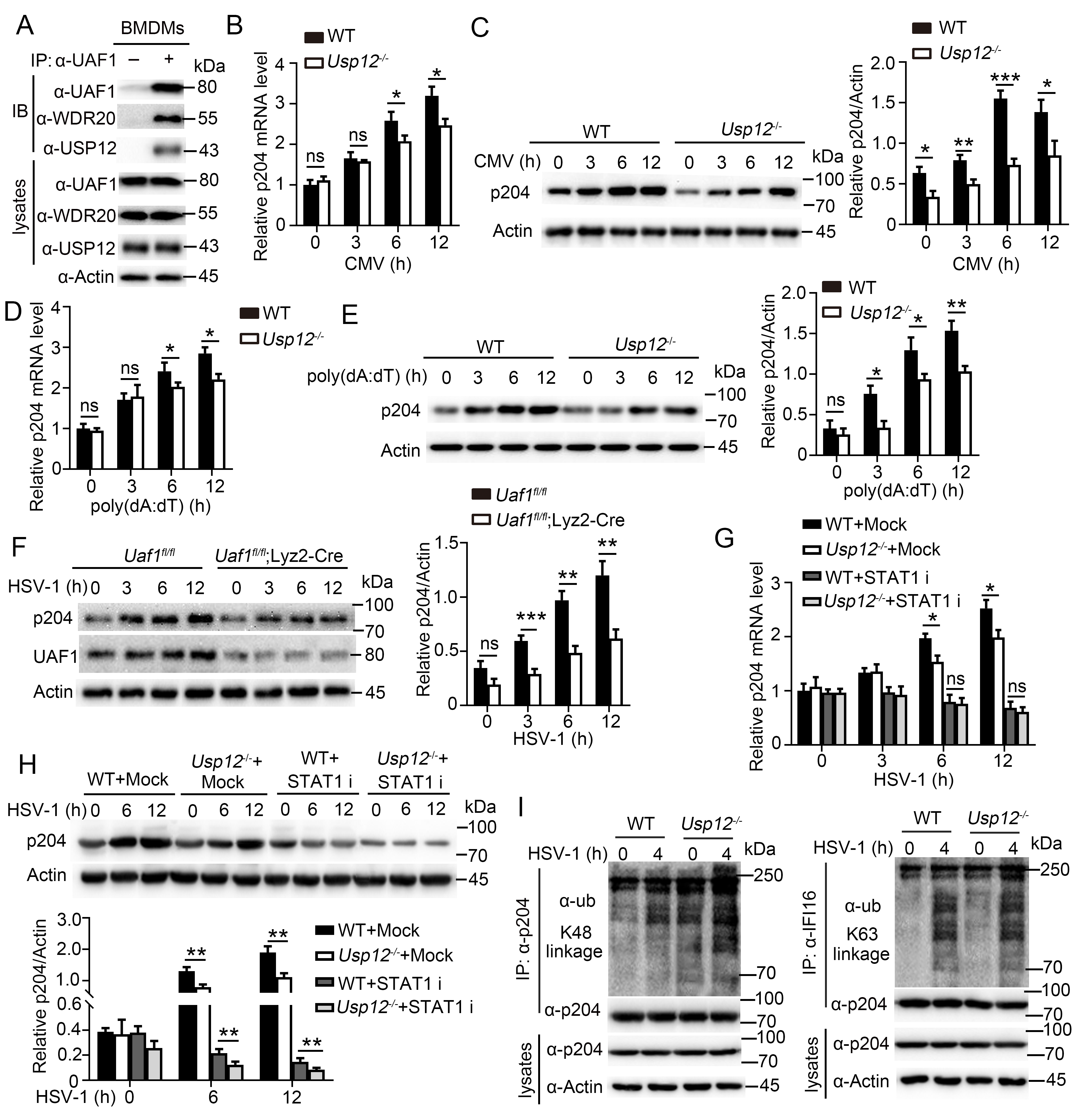

Supplement: S1 Fig — (A) WT BMDMs were infected HSV-1 for 6 hours followed by immunoprecipitation (IP) using anti-UAF1 or IgG, and immunoblotting (IB) analysis. (B-C) WT and Usp12-/- BMDMs were infected with CMV for the indicated times. p204 expression levels were detected by qPCR (B) or western blot (C). Densitometry quantification of band intensity are presented in the right panel. (D-E) WT and Usp12-/- BMDMs were stimulated with poly(dA:dT) for the indicated times. p204 expression levels were detected by qPCR (D) or western blot (E). Densitometry quantification of band intensity are presented in the right panel. (F) Uaf1fl/fl and Uaf1fl/fl;Lyz2-Cre BMDMs were infected with HSV-1 for the indicated times. p204 expression levels were detected by western blot. Densitometry quantification of band intensity are presented in the right panel. (G-H) WT and Usp12-/-BMDMs were pretreated with STAT1 inhibitor Fludarabine (STAT1 i), and infected with HSV-1 for indicated time. p204 expression levels were detected by qPCR (G) or western blot (H). Densitometry quantification of band intensity are presented in the below panel. (I) p204 IB and K48 and K63 ubiquitination analysis using whole-cell extracts of WT and Usp12-/- BMDMs infected with HSV-1 for the indicated time. Data shown are the mean ±SD. *P < 0.05, and **P < 0.01. Ns, no significant. Data are representative of three independent experiments with similar results. (TIF) [file ppat.1011827.s005.tif]

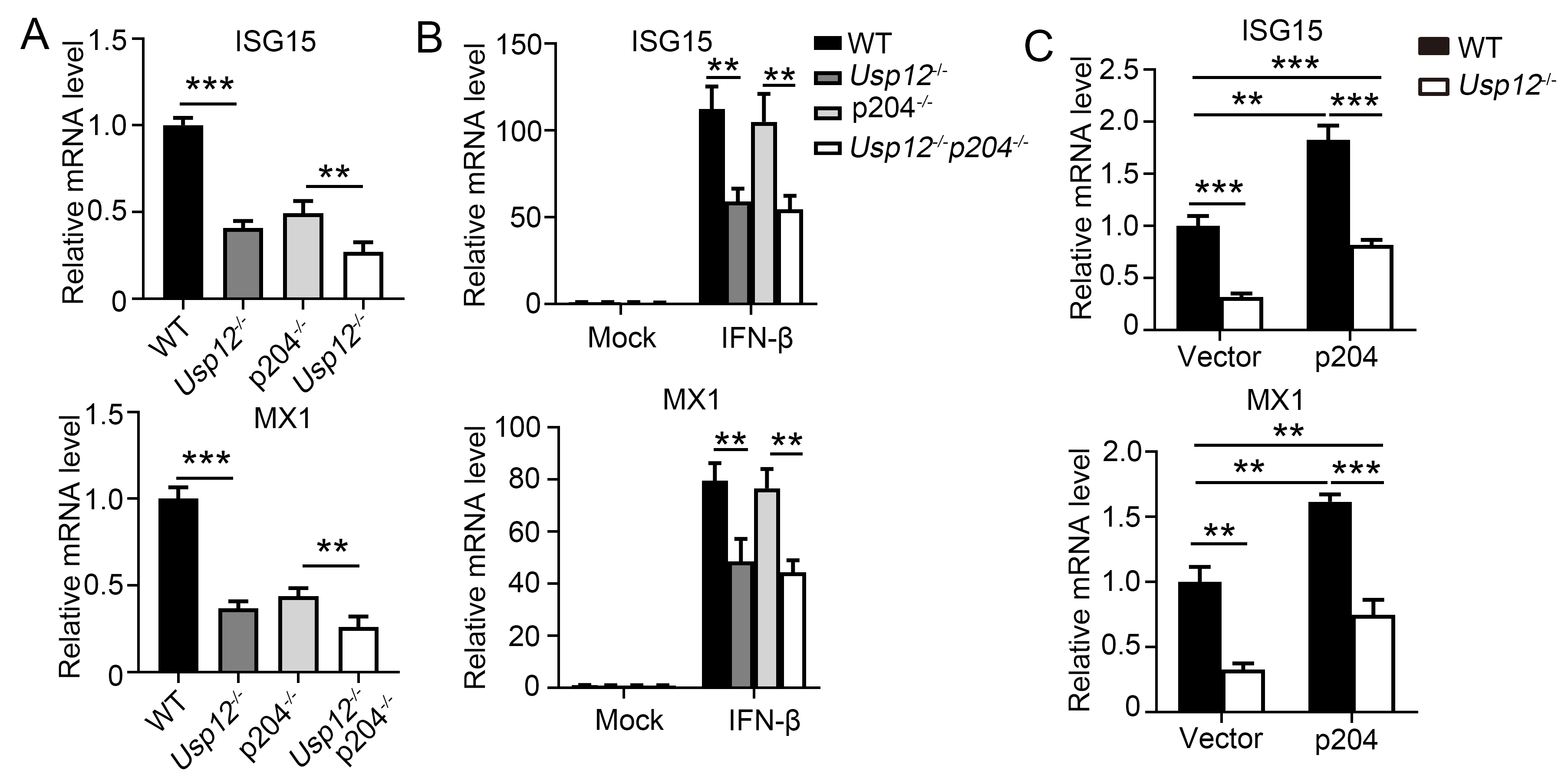

Supplement: S2 Fig — (A) WT, Usp12-/-, p204-/- and Usp12-/-p204-/- BMDMs were infected with HSV-1 for 24 hours, and expression of ISG15 and MX1 was determined by qPCR. (B) WT, Usp12-/-, p204-/- and Usp12-/-p204-/- BMDMs were stimulated with IFN-β for 6 hours. Expression of ISG15 and MX1 was determined by qPCR. (C) WT or Usp12-/- BMDMs were transfected with control or expression vector for p204, and infected with HSV-1 for 24 hours. Expression of ISG15 and MX1 was determined by qPCR. Data shown are the mean ±SD. **P < 0.01 and ***P < 0.001 by an unpaired t-test. Ns, no significant. Data are representative of three independent experiments with similar results. (TIF) [file ppat.1011827.s006.tif]
